# Estrogenic, androgenic, and genotoxic activities of zearalenone and deoxynivalenol in *in vitro* bioassays including exogenous metabolic activation

**DOI:** 10.1007/s12550-024-00529-2

**Published:** 2024-04-08

**Authors:** Maria Yu, Agneta Oskarsson, Jan Alexander, Johan Lundqvist

**Affiliations:** 1 Department of Biomedical Sciences and Veterinary Public Health, Swedish University of Agricultural Sciences, Box 7028, SE-750 07 Uppsala, Sweden; 2 Norwegian Scientific Committee for Food and Environment, Norwegian Institute of Public Health, P.O. Box 222 Skøyen, NO-0213 Oslo, Norway

**Keywords:** Deoxynivalenol, Effect-based methods, Endocrine receptors, *In vitro* bioassays, Metabolism, Micronuclei formation, Zearalenone

## Abstract

Zearalenone (ZEN) and deoxynivalenol (DON) and their derivatives are well-known mycotoxins, which can occur not only in crops but also in water bodies, including drinking water sources. *In vitro* bioassays can be used to detect biological effects of hazardous compounds in water. To this, when studying biological effects and toxicity *in vitro*, metabolism is important to consider. In this study, ZEN, α-zearalenol (α-ZEL), DON, 3-acetyl DON, and 15-acetyl DON were evaluated *in vitro* for hormone receptor–mediated effects (estrogen receptor [ER] and androgen receptor [AR]) and genotoxicity (micronucleus assay) in the presence of an exogenous metabolic activation system (MAS). The ER bioassay proved to be a highly sensitive method to detect low concentrations of the ZEN compounds (EC_10_ values of 31.4 pM for ZEN, 3.59 pM for α-ZEL) in aqueous solutions. In the presence of the MAS, reduced estrogenic effects were observed for both ZEN compounds (EC_10_ values of 6.47 × 10^3^ pM for ZEN, 1.55 × 10^2^ pM for α-ZEL). Of the DON compounds, only 3-acetyl DON was estrogenic (EC_10_ of 0.31 µM), and the effect was removed in the presence of the MAS. Anti-androgenic effects of the ZEN compounds and androgenic effects of the DON compounds were detected in the micromolar range. No induction of genotoxicity was detected for ZEN or DON in the presence of the MAS. Our study highlighted that inclusion of exogenous MAS is a useful tool to detect biological effects of metabolites in *in vitro* bioassays.

**Supplementary Information:**

The online version contains supplementary material available at 10.1007/s12550-024-00529-2.

## Introduction

The genus *Fusarium* is a group of so-called field fungi that grow on field crops and produce several types of mycotoxins including trichothecenes and zearalenone (D’Mello et al. 1999). The contamination of food crops and feed with such mycotoxins occurs at a global scale, and their adverse effects on human and animal health are significant concerns (Beltman et al. 2000; Fung and Clark 2004; Rai et al. 2020). Deoxynivalenol (DON) and its derivatives, 3-acetyl DON (3-Ac-DON) and 15-acetyl DON (15-Ac-DON), are trichothecenes commonly found in various food commodities (Kamle et al. 2022; Mishra et al. 2020). Zearalenone (ZEN) and its metabolites, such as α-zearalenol (α-ZEL), are another group of common food contaminants (Ropejko and Twarużek 2021). While the occurrence of these mycotoxins in food crops and feed has been extensively studied, increasing attention has been given to the fact that fungi and mycotoxins also occur in surface and drinking waters (Al-Gabr et al. 2014; Bucheli et al. 2008; Gromadzka et al. 2009; Hageskal et al. 2009; Jaro et al. 2015; Kolpin et al. 2014; Oliveira et al. 2018; Székács 2021). ZEN, for instance, has been reported at concentrations ranging from 0.5 up to 80.6 ng/L in different surface water types (Bucheli et al. 2008; Gromadzka et al. 2009; Maragos 2012; Waśkiewicz et al. 2012). Mycotoxins in water partly originate from leaching from infected crop fields and soil or the growth of fungi on crop residues (Al-Gabr et al. 2014; Hartmann et al. 2008; Schenzel et al. 2012; Waśkiewicz et al. 2012). Mycotoxins have also been detected in water distribution systems (Kelley 2003; Mhlongo et al. 2019; Russell and Paterson 2007), in relation to biofilm formation (Siqueira et al. 2011; Steven 2000), in wastewater treatment plant effluents (Kolpin et al. 2014), and can occur following prolonged storage of water in cisterns/reservoirs (Hageskal et al. 2009).

Effect-based methods using *in vitro* bioassays are recommended for water quality monitoring and detection of effects from hazardous organic micropollutants (Brack et al. 2019; Enault et al. 2023; Macova et al. 2011). As metabolism can impact the toxicity of micropollutants, such as these mycotoxins, it is important to consider their metabolic fate and thus possibilities of activation/detoxification (Boevre et al. 2015). For instance, ZEN and DON and their metabolites can cause agonistic and/or antagonistic endocrine effects (Demaegdt et al. 2016; Frizzell et al. 2011; Ndossi et al. 2012). In particular, ZEN and one of its main metabolites, α-ZEL, are recognized for their potent estrogenic effects (Metzler et al. 2010) and affinity for mammalian estrogen receptors (ERs) (Andres et al. 2013; Tatay et al. 2018). In comparison, the endocrine effects of DON and its metabolites have been less studied. Other important health-relevant effects of these mycotoxins, such as genotoxicity, have also been reported in various experimental models, as highlighted by the European Food Safety Authority’s (EFSA) CONTAM Panel (EFSA 2011, 2016, 2017a, b). Studies that integrate metabolism into effect-based *in vitro* methods are particularly critical to include relevant toxicity pathways in hazard assessments. However, many of the genetically engineered cell lines used in effect-based *in vitro* studies have a limited capacity for xenobiotic metabolism/biotransformation due to a lack of the necessary enzymes. The incorporation of exogenous metabolic components into reporter gene assays has already been attempted by a limited number of *in vitro* studies for other endocrine-disrupting compounds (Charles et al. 2000; Jacobs et al. 2008; Mollergues et al. 2017; van Vugt-Lussenburg et al. 2018; Yoshihara et al. 2001), but to the best of our knowledge, it has not yet been attempted with these mycotoxins.

In the current study, the hypothesis that metabolism has an impact on the hormonal receptor and genotoxic activities of ZEN, DON, and their primary derivatives was explored. The influence of exogenous metabolic components on the effects of ZEN, DON, and their primary derivatives to the estrogen and androgen receptors was investigated using *in vitro *reporter gene assays in mammalian cell lines. The exogenous metabolic components consisted of rat hepatic S9 fractions supplemented with phase I and/or phase II cofactors. The genotoxic effects of ZEN and DON on micronuclei (MN) formations in the human lymphoblastic TK6 cell line were also assessed without and with exogenous metabolic components.

## Materials and methods

### Mycotoxin compounds

Biopure^™^ Solid Standards of zearalenone (CAS 17924-92-4), deoxynivalenol (CAS 51481-10-8), 3-acetyl deoxynivalenol (CAS 50722-38-8), and 15-acetyl deoxynivalenol (CAS 88337-96-6) were purchased from Romer Labs (Diagnostic GmbH, Tulln, Austria). α-Zearalenol (CAS 36455-72-8), ≥ 98.0% purity, powder form was purchased from Sigma-Aldrich (Germany). Chemical structures of all test compounds are provided in the supplementary information (Fig. SI-1). All compounds were dissolved in dimethyl sulfoxide (DMSO, ≥ 99.9%, CAS 67-68-4) purchased from Sigma-Aldrich (Germany) to prepare stock solutions.

For the reporter gene assays, the metabolic activation components consisted of a combination of rat liver S9 fractions, phase I, and phase II cofactors. Liver S9 fractions from rats induced with a mixture of β-naphtoflavone and phenobarbital (βNF/PB) were purchased from Xenometrix (Allschwil, Switzerland). For the phase I cofactors, an NADPH regeneration system consisting of substrate 26 mM NADP^+^, 66 mM glucose-6-phosphate (G-6-P) (20 × concentrate), 66 mM MgCl_2_, and 40 U/mL glucose-6-phosphate dehydrogenase (100 × concentration) was purchased from Promega Biotech AB (Nacka, Sweden). For the phase II cofactors, a mixture was prepared in-house consisting of reduced l-glutathione (GSH), uridine 5-diphospho-glucuronic acid (UDPGA), and 3′-phosphoadenosine 5′-phosphosulfate lithium salt (PAPS). All three phase II components were purchased from Sigma-Aldrich, Germany.

For the micronucleus (MN) assay, an S9 Cofactor Kit (Xenometrix, art. no. PCO-0800) was purchased to supplement the S9 fractions as this kit was relevant to the assay. It is a ready-to-use kit containing buffer salts, G-6-P, and NADP^+^ to be added when preparing the S9 mix for metabolic activation studies.

### *In vitro* bioassays

In the following, brief descriptions of the cell lines tested and bioassays used are provided. The concentration ranges studied for the ZEN compounds were between 5.0 × 10^−8^ and 50 µM in the ER agonistic and antagonistic modes and between 0.78 and 25 µM in the AR agonistic and antagonistic modes. The concentration ranges studied for the DON compounds were between 2.5 × 10^−2^ and 3.2 µM in the ER agonistic and antagonistic modes and between 1.3 × 10^−2^ and 1.6 µM in the AR agonistic and antagonistic modes. More detailed descriptions of all cell lines and maintenance as well as the bioassays are provided in the supplementary information (SI-1).

#### Luciferase reporter gene assays

Estrogen receptor (ER) activities were assayed in the human breast carcinoma cell line MCF-7, stably transfected with an estrogen receptor–sensitive luciferase plasmid (VM7Luc4E2 cells). The cells were kindly donated by the late Professor Michael Denison (University of California, USA). Androgen receptor (AR) activities were assayed in the Chinese hamster ovary cell line (CHO) stably transfected with an androgen receptor responsive luciferase plasmid and an expression vector for the human androgen receptor (AR-EcoScreen GR KO M1). The cells were obtained from the JCRB (JCRB1761). In all assays, cells were plated in white 384-well plates (Costar^®^ Corning Incorporated) in assay media and incubated at 37 °C and 5% CO_2_ for 24 h. Thereafter, the cells were exposed to serial dilutions of the test compounds and incubated for another 24 h. Following that, the supernatant was removed from the plates, and cells were lysed with 10 µL passive lysis buffer (Promega, Southampton, UK) after which the plates were shaken for 15–30 min. Luciferase activity was measured using the Luciferase^®^ Reporter Assay System (Promega) according to the manufacturer’s instructions. Luminescence was measured on a Spark^®^ Multimode Microplate Reader with an automatic injection syringe. The injection volume for the firefly luciferase reagent in the activity assays was 10 µL/well.

#### Cell viability assays

Cell viabilities of both cell lines in the presence of the test compounds alone as well as with exogenous metabolic components were measured using the ATP assay (CellTiter-Glo^®^ Luminescent Cell Viability Assay, Promega, USA). The concentration ranges studied for the ZEN compounds were between 6.3 × 10^−4^ and 10 µM in the ER assays and between 0.79 and 50 µM in the AR assays. The concentration ranges studied for the DON compounds were between 5.0 × 10^−6^ and 5.0 µM in the ER assays and between 1.3 × 10^−2^ and 1.6 µM in the AR assays. The plating densities and incubation periods were the same as for the activity assays for both cell lines, and all were plated in white 384-well plates (Costar^®^ Corning Incorporated). Following incubation with the test compounds, 25 µL of CellTiter-Glo^®^ Reagent (Promega) was added to each well, and the plates incubated for approximately 25 min prior to plate readings. Luminescence was measured on a Spark^®^ Multimode Microplate Reader.

#### Assay controls and reference compounds

For the ER agonist and antagonist assays, 0.36–367 pM of 17β-estradiol (E2) and 0.19–24.5 pM of raloxifene (RAL) were included as reference compounds, respectively. For the AR agonist and antagonist assays, 0.001–1000 nM of dihydrotestosterone (DHT) and 0.01–10000 nM of hydroxy flutamide (OHF) were used, respectively. Vehicle controls consisting of 1% v/v DMSO, equivalent to the DMSO plate concentration of the dissolved test compounds, were included in all assays. For the ER and AR antagonist assays, test compounds were incubated in medium spiked with 9.2 × 10^−5^ µM of E2 and 2.0 × 10^−4^ µM of DHT, respectively, to induce receptor activity.

#### Incubation with exogenous metabolic activation system (MAS)

For the ER and AR assays, the exogenous MAS consisted of a mix of S9 solutions in assay medium containing 0.07 mg/mL S9 fractions along with 0.1 × NADP^+^, glucose-6-phosphate, and glucose-6-phosphate dehydrogenase constituting the Phase I (PHI) cofactors and 100 mM GSH, 25 mM UDPGA, and 0.1 mM PAPS constituting the Phase II (PHII) cofactors. The test compounds, vehicle controls, and reference compounds were tested in different combinations of the S9 mixes: S9 with only PHI cofactors for phase I metabolism (only for ZEN compounds), S9 with PHI and II cofactors for phase I + II metabolism, and S9 with only PHII cofactors for phase II metabolism (only for DON compounds, as they are mainly metabolized by phase II reactions). Test compounds, vehicle controls, and reference compounds were also tested with only the S9 fraction to monitor if the effects seen were due to protein binding of the test compounds to the S9 fraction proteins rather than metabolism. For the agonist assays, the cells were co-incubated with the test compounds and the MAS mixes for 24 h. For the antagonist assays, to prevent the agonists in the spiking medium from being metabolized, the test compounds were first pre-incubated with the metabolic mixes at 37 °C for 75 min, then inactivated at 55 °C for 15 min thereafter. Following this, the test compounds along with the spiked medium were added to the plated cells (the medium was first removed from the plates) and the plates were incubated for 24 h.

#### Micronucleus assay

Genotoxicity was assessed in the human lymphoblastic TK6 cell line (American Type Culture Collection, ATCC, Manassas, VA, USA) using the *in vitro* micronucleus (MN) assay. The average doubling time of the TK6 cells was 10–12 h. The assay was conducted in general accordance with the OECD guideline no. 487 (OECD 2016) and followed the treatment schedule described in the guideline for the evaluation of experimental conditions with and without metabolic activation. Briefly, for the long-term exposure, cells were continuously exposed to the test compounds for 24 h without metabolic activation. For the short-term exposure, cells were exposed to the test compounds without and with metabolic activation for 5 h. For the short-term exposure with metabolic activation, the cells were co-incubated with the S9 fractions and the S9 cofactor kit. The cofactor solution was prepared according to the instructions provided by the manufacturer (Xenometrix) to obtain a final S9 concentration of 3%. Thereafter, the cells were collected by centrifugation, the treatment medium was removed, the cells rinsed, and the wells replaced with fresh experimental medium. The plates were then incubated for the remainder of the 24-h incubation period. Vehicle (DMSO) controls, along with mitomycin C (100 and 200 nM) and benzo[a]pyrene (10 and 15 µM), were included as the positive controls for the long-term (24 h) and short-term (5 h) exposures, respectively. Following treatment with the test compounds, the cells were stained for flow cytometry analysis using a MicroFlow^®^ Kit (Litron Laboratories, Rochester, NY, USA) via a two-color sequential staining technique with ethidium monoazide (EMA) and then SYTOX Green. The flow cytometry analysis was performed using a FACSVerse (BD Biosciences, Franklin Lakes, NJ, USA). From each test concentration, approximately 20,000 events were collected. Further information regarding the assay set-up and flow cytometry analysis is provided in the supplementary information (SI-1).

### Data analyses

All experiments were performed in quadruplicate (e.g., technical replicates) for the cell viability and ER/AR activity assays and in triplicate for the MN assay, and a minimum of two independent experiments (i.e., inter-assay replication) were performed to confirm no assay drift or bias over time. For the cell viability assays, luminescence signals of the test concentration replicates were first normalized to the mean of the vehicle controls, set at 1.00, and cytotoxicity was defined as <0.80 of the mean luminescence of the vehicle controls. To quantify the hormone receptor–mediated induction of the luciferase gene in the agonistic version of the ER and AR assays, the mean response of the vehicle controls was first subtracted from all test concentration replicates. All adjusted values were then normalized to the mean response of the vehicle controls and then to the mean maximum response of the highest concentration of the respective reference compound (assay maximum, set to 100%). Concentration-effect curves (CECs) were then generated from the normalized data using the software GraphPad Prism (v. 10.1.1) and non-linear sigmoidal regression was performed to determine the concentrations causing a 10% effect (EC_10_) for each test compound. For the antagonistic version of the two assays, the mean response of the unspiked vehicle control was first subtracted from the test concentration replicates. All adjusted values were then normalized to the mean response of the unspiked vehicle control and then to the mean response of the spiked vehicle control. Antagonistic activity was defined as a decrease in activity compared to the spiked vehicle control. CECs were generated in GraphPad to determine the concentrations causing a 30% inhibitory effect (IC_30_) for each test compound. To compare the EC and IC values of the test compounds without and with the exogenous MAS, the 95% confidence intervals of the EC/IC values generated by GraphPad from the non-linear sigmoidal regressions of the data were used. For the ER and AR ago assays, cut-off levels for positive responses in activities were defined as 10% of the assay maximum and referred to as the limit of detection (LOD). Any result for which the EC value was below 10% of the assay max was then regarded as “ <LOD”. For the cut-off levels in the ER and AR anta assays, any result for which the IC value was above 30% of the assay max was regarded as “ <LOD”.

For the MN assay, the mean number of micronuclei (% MN) obtained from the flow cytometry analysis for each test concentration was compared to that of the vehicle control using a one-way ANOVA comparison followed by Dunnett post-hoc test function in the GraphPad software. Genotoxicity was defined as a statistically significant increase in the mean % MN compared to that of the vehicle control at the 95% confidence level, and a *p*-value of <0.05 was considered significant. A comparison of the results without and with the metabolic components was also evaluated using the one-way ANOVA comparison followed by Dunnett post hoc test. To control for non-specific effects due to general cytotoxicity, a <4-fold EMA-positive event increase over the vehicle control was applied as a cytotoxicity limit.

## Results

### Concentration-range finding trials

Initial trials with only the test compounds (i.e., without exogenous MAS) were conducted to determine suitable non-cytotoxic concentration ranges. The results of the cytotoxicity assessments for the selected concentration ranges are provided in the supplementary information (Fig. SI-2). For the ER and AR assays, CECs of the activities were generated for each compound. EC_10_ and IC_30_ values are summarized in Table 1 and graphs of the CECs are presented in the supplementary information (Figs. SI-3 & SI-4). Results from the MN assay are presented further below.

**Table 1:** Summary of effect concentrations (EC_10_ for receptor agonism and IC_30_ for receptor antagonism) of the test compounds in the estrogen receptor (ER) and androgen receptor (AR) assays without an exogenous metabolic activating system (MAS)

**Test compound**	**ER agonism**	**ER antagonism**	**AR agonism**	**AR antagonism**
	**EC**_**10**_** (pM)**	**IC**_**30**_** (µM)**	**EC**_**10**_** (µM)**	**IC**_**30**_** (µM)**
Zearalenone (ZEN)	34.07	2.85	<LOD	3.30
α-Zearalenol (α-ZEL)	3.59	1.39	<LOD	0.88
Deoxynivalenol (DON)	<LOD	0.91	0.83	<LOD
3-Acetyl deoxynivalenol (3-Ac-DON)	310,000	<LOD	1.31	<LOD
15-Acetyl deoxynivalenol (15-Ac-DON)	<LOD	0.99	0.77	<LOD

*LOD* limit of detection

In the initial concentration-range finding trials, high ER agonistic activities (i.e., in the pM range) were detected in both ZEN compounds. Some ER agonistic activity was detected in 3-Ac-DON, but none for DON or 15-Ac-DON. Also, ER antagonistic activities were detected in both ZEN compounds (albeit with considerably lower potency than for ER agonism, in the µM range) as well as for DON and 15-Ac-DON. No ER antagonism was detected for 3-Ac-DON. No AR agonistic activities were detected in either of the ZEN compounds, while AR anta activities were observed (in the µM range). As well, no AR antagonistic activities were detected in any of the DON compounds, while AR ago activities were observed (in the µM range).

### Effect of exogenous metabolic activating systems in hormone receptor assays

The results of the activities for all test compounds in the ER and AR assays without and with the exogenous MAS are presented in the subsections below as CECs. Summary tables of the calculated effect concentrations and 95% confidence intervals are also presented in Tables 2 and 3. To verify that the exogenous MAS metabolic components themselves did not induce cytotoxicity, cell viabilities in the presence of the tested MAS concentrations were also assessed. No cytotoxicity was observed and the results are provided in the Supplementary Information (Figs. SI-5 & SI-6).

**Table 2:** Summary of effect concentrations (EC_10_ for receptor agonism and IC_30_ for receptor antagonism) of the ZEN compounds in the ER and AR assays without and in the presence of an exogenous MAS

**Test compound**	**MAS**	**ER agonism** **EC**_**10**_** (pM)**				**ER antagonism** **IC**_**30**_** (µM)**				**AR agonism** **EC**_**10**_** (pM)**				**AR antagonism** **IC**_**30**_** (µM)**			
	S9	** − **	** + **	** + **	** + **	** − **	** + **	** + **	** + **	** − **	** + **	** + **	** + **	** − **	** + **	** + **	** + **
	+ PHI	** − **	** − **	** + **	** + **	** − **	** − **	** + **	** + **	** − **	** − **	** + **	** + **	** − **	** − **	** + **	** + **
	+ PHII	** − **	** − **	** − **	** + **	** − **	** − **	** − **	** + **	** − **	** − **	** − **	** + **	** − **	** − **	** − **	** + **
ZEN		46.5 [29.5,72.4]	63.3 [56.2,97.2]	984 [740,1 279]	6 472 [4 121,9 506]	2.92 [2.09,3.89]	3.41 [2.40,4.68]	10.3 [8.71,11.8]	16.2 [6.76,16.2]	<LOD	<LOD	<LOD	<LOD	2.37 [1.95,2.75]	2.14 [1.82,2.45]	7.33 [6.31,8.13]	18.0 [13.8,23.4]
α-ZEL		3.70 [2.51,5.37]	4.24 [2.82,6.31]	49.3 [30.2,75.9]	155.1 [93.3,245.4]	1.03 [0.33,1.78]	1.75 [0.69,3.02]	2.42 [1.35,3.63]	5.10 [2.88,8.13]	<LOD	<LOD	<LOD	<LOD	0.95 [0.85,1.05]	0.74 [0.62,0.87]	1.12 [0.91,1.32]	1.61 [1.10,2.14]

95% confidence intervals [lower limit or LL, upper limit or UL] of the EC_10_/IC_30_ values are presented in brackets below each value

**Table 3:** Summary of effect concentrations (EC_10_ for receptor agonism and IC_30_ for receptor antagonism) of the DON compounds in the ER and AR assays without and in the presence of exogenous MAS

**Test compound**	**MAS**	**ER agonism** **EC**_**10**_** (µM)**				**ER antagonism** **IC**_**30**_** (µM)**				**AR agonism** **EC**_**10**_** (µM)**				**AR antagonism** **IC**_**30**_** (µM)**			
	S9	** − **	** + **	** + **	** + **	** − **	** + **	** + **	** + **	** − **	** + **	** + **	** + **	** − **	** + **	** + **	** + **
	+ PHI	** − **	** − **	** + **	** − **	** − **	** − **	** + **	** − **	** − **	** − **	** + **	** − **	** − **	** − **	** + **	** − **
	+ PHII	** − **	** − **	** + **	** + **	** − **	** − **	** + **	** + **	** − **	** − **	** + **	** + **	** − **	** − **	** + **	** + **
DON		<LOD	<LOD	<LOD	<LOD	0.81 [0.69,1.00]	0.75 [0.54,1.05]	0.37 [0.25,0.58]	0.25 [0.18,0.38]	0.87 [0.81,0.93]	0.84 [0.72,0.98]	0.45 [0.39,0.52]	0.38 [0.32,0.46]	<LOD	<LOD	<LOD	<LOD
3-Ac-DON		0.45 [0.41,0.49]	0.53 [0.49,0.59]	<LOD	<LOD	<LOD	<LOD	<LOD	<LOD	1.11 [0.95,1.26]	1.07 [0.91,1.23]	0.91 [0.65,1.35]	0.63 [0.52,0.78]	<LOD	<LOD	<LOD	<LOD
15-Ac-DON		<LOD	<LOD	<LOD	<LOD	1.09 [0.98, 1.20]	1.11 [0.81,1.38]	0.48 [0.30,0.72]	0.24 [0.18,0.32]	0.81 [0.74,0.87]	0.70 [0.62,0.76]	0.52 [0.45,0.62]	0.50 [0.45,0.56]	<LOD	<LOD	<LOD	<LOD

95% confidence intervals [LL, UL] of the EC_10_/IC_30_ values are presented in brackets below each value

#### ZEN compounds—estrogen receptor agonism and antagonism

**Fig. 1:**
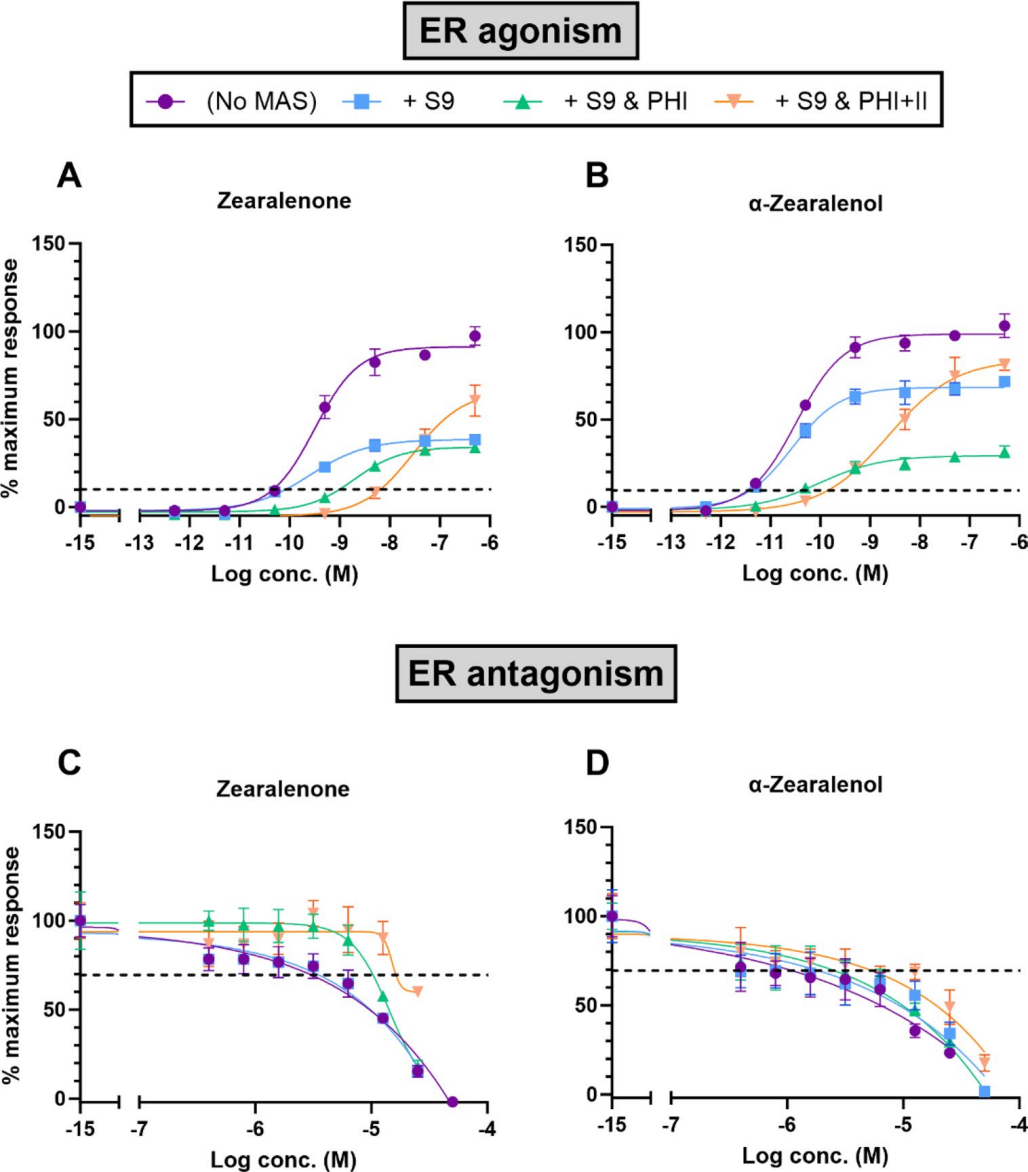
CECs of the ER agonistic and antagonistic effects of ZEN (**A**, **C**) and α-ZEL (**B**, **D**) in the presence of exogenous MAS. Each test compound (*n* = 4) was assayed in the absence of MAS (purple, circles), in the presence of S9 alone (light blue, squares), S9 with PHI cofactors (green, triangles), or S9 with PHI and PHII cofactors (light orange, inverted triangles). Data presented as mean ± SD

The ER agonistic effects of both ZEN compounds were reduced in the presence of S9 with the PHI cofactors alone and most in the presence of S9 with the PHI and II cofactors (Fig. 1A and B, Table 2). At higher concentrations of the ZEN compounds, S9 alone reduced the ER agonistic activities, although at EC_10_, the effect was not significant (i.e., overlap in the 95% confidence intervals [95% CIs], Table 2), while phase I metabolism caused a significant reduction in ER activities, which was further reduced by phase II metabolism (Table 2). A reduction of ER agonistic activity after treatment of cells with only S9 was also seen at higher concentrations of E2 (Fig SI-7A), indicating binding of E2 to S9 proteins. The ER antagonistic activity of ZEN and α-ZEL was reduced after phase II metabolism, while S9 alone or phase I metabolism did not cause any significant effects (Fig. 1C and D, Table 2).

#### ZEN compounds—androgen receptor agonism and antagonism

**Fig. 2:**
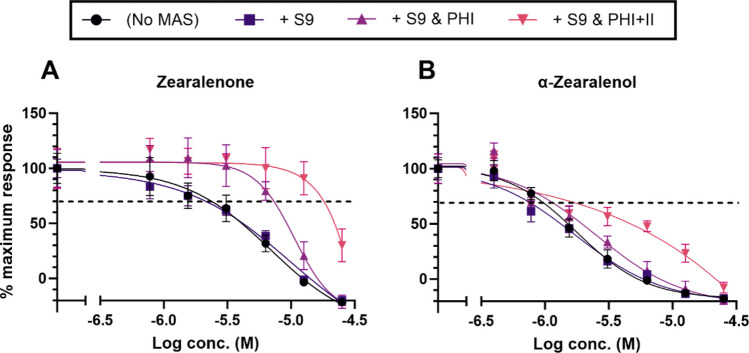
CECs of the AR antagonistic effects of ZEN (**A**) and α-ZEL (**B**) in the presence of exogenous MAS. Each test compound (*n* = 4) was assayed in the absence of MAS (black, circles), in the presence of S9 alone (dark purple, squares), S9 with PHI cofactors (purple, triangles), or S9 with PHI and PHII cofactors (pink, inverted triangles). Data presented as mean ± SD

Despite detecting no AR agonistic effects from either of the two ZEN compounds in the initial trials, both compounds were tested again in the presence of exogenous MAS to determine if the MAS had any metabolically activating effects. However, no effects were detected. For antagonism, there was no effect from S9 alone. The antagonistic effects of ZEN decreased in the presence of S9 with PHI cofactors and further in the presence of PHI and II cofactors (Fig. 2A, Table 2). For α-ZEL, the antagonistic effects decreased mostly in the presence of S9 with PHI and II cofactors (Fig. 2B, Table 2).

#### DON compounds—estrogen receptor agonism and antagonism

**Fig. 3:**
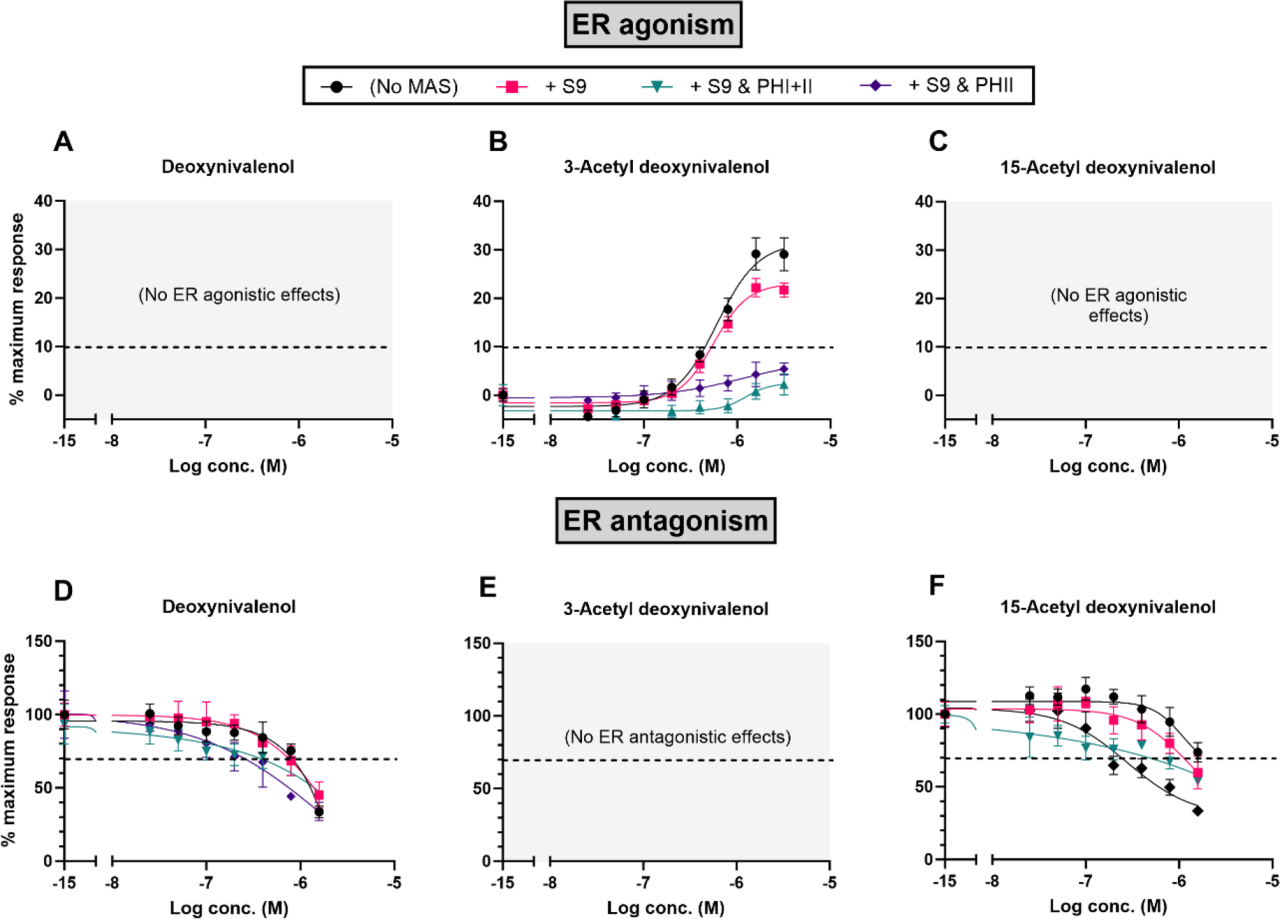
CECs of the ER agonistic and antagonistic effects of DON (**A**, **D**), 3-Ac-DON (**B**, **E**), and 15-Ac-DON (**C**, **F**) in the presence of exogenous MAS. Each test compound (*n* = 4) was assayed in the absence of MAS (black, circles), in the presence of S9 alone (pink, squares), S9 with PHI and PHII cofactors (teal, inverted triangles), or S9 with PHII cofactors (dark purple, diamonds). Data presented as mean ± SD

For the DON compounds, as observed in the initial trials, no ER agonistic effects of DON and 15-Ac-DON (Fig. 3A, C) or antagonistic effects of 3-Ac-DON (Fig. 3E) were detected. The exogenous MAS had no effect either. For 3-Ac-DON, the ER agonistic effects appeared to be completely deactivated (i.e., the highest detected activity was below 10% of the maximum response) both in the presence of S9 with the PHI and II cofactors and in the presence of S9 with the PHII cofactors alone (Fig. 3B, Table 3). The ER antagonistic effects of DON and 15-Ac-DON both increased in the presence of S9 with the PHII cofactors alone (Fig. 3D, F, Table 3). While increased antagonistic effects were also observed in the presence of S9 with the PHI and II cofactors, there was a slight overlap in the 95% CIs for DON in the presence of S9 alone and S9 with PHI and II cofactors (Table 3).

#### DON compounds—androgen receptor agonism and antagonism

**Fig. 4:**
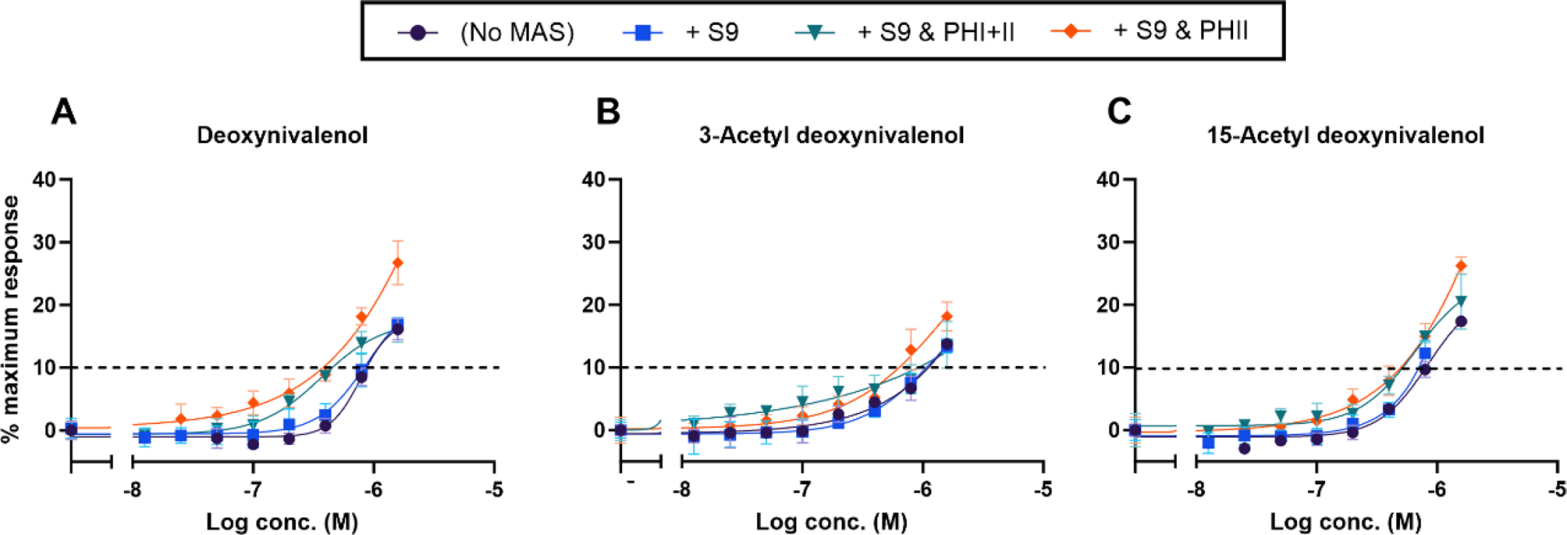
CECs of the AR agonistic effects of DON (**A**), 3-Ac-DON (**B**), and 15-Ac-DON (**C**) in the presence of exogenous MAS. Each test compound (*n* = 4) was assayed in the absence of MAS (dark purple, circles), in the presence of S9 alone (light blue, squares), S9 with PHI and PHII cofactors (teal, inverted triangles), or S9 with PHII cofactors (orange, diamonds). Data presented as mean ± SD

For all three DON compounds, the agonistic effects increased in the presence of S9 with the PHII cofactors alone (Fig. 4A–C, Table 3). The presence of S9 with the PHI and II cofactors also increased the agonistic effects of DON and 15-Ac-DON, while only slightly for 3-Ac-DON (i.e., the upper limit of the 95% CI for 3-Ac-DON in the presence of S9 with the PHI and II cofactors overlapped the 95% CI’s for the test compound alone and also with S9 alone).

### Genotoxicity

#### Genotoxicity of ZEN and DON (without MAS)

**Fig. 5:**
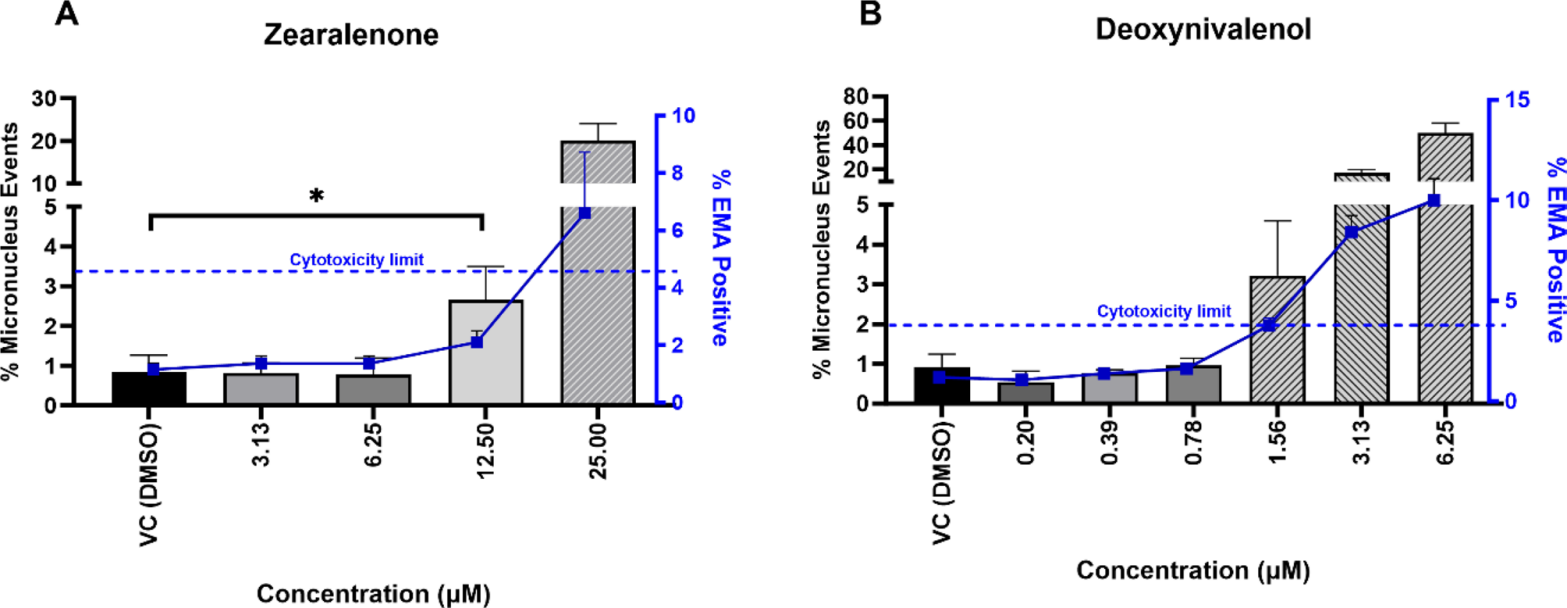
Micronuclei formations (left y-axis) and cytotoxicity (right y-axis) in TK6 cells following continuous long-term exposure (24 h) to ZEN (**A**), and DON (**B**). For micronuclei formations, data bars for each test concentration presented as mean ± SD (*n* = 3). Treatment groups were compared to the vehicle control via pair-wise comparisons. Asterisk (*) represents the level of significant difference in the % micronucleus events from the vehicle control (*p* ≤ 0.001). For cytotoxicity, the scoring criteria was set as 4-fold increase in % ethidium monoazide (EMA)-Positive over the vehicle control. Blue square symbols of the %EMA-Positive results for each test concentration presented as mean ± SD

The genotoxic effects of ZEN and DON in the TK6 cell line were assessed using the MN assay without MAS for a 24-h incubation period. Concentration-range finding trials were conducted to determine non-cytotoxic concentration ranges. The results are shown in Fig. 5. No statistically significant increases in % MN were detected in any of the non-cytotoxic test concentrations for DON compared to the vehicle control. For ZEN, a statistically significant increase in % MN was detected at the highest non-cytotoxic test concentration (12.5 µM) compared to the control (*p*-value of 0.0007).

#### Effect of exogenous MAS in the MN assay

**Fig. 6:**
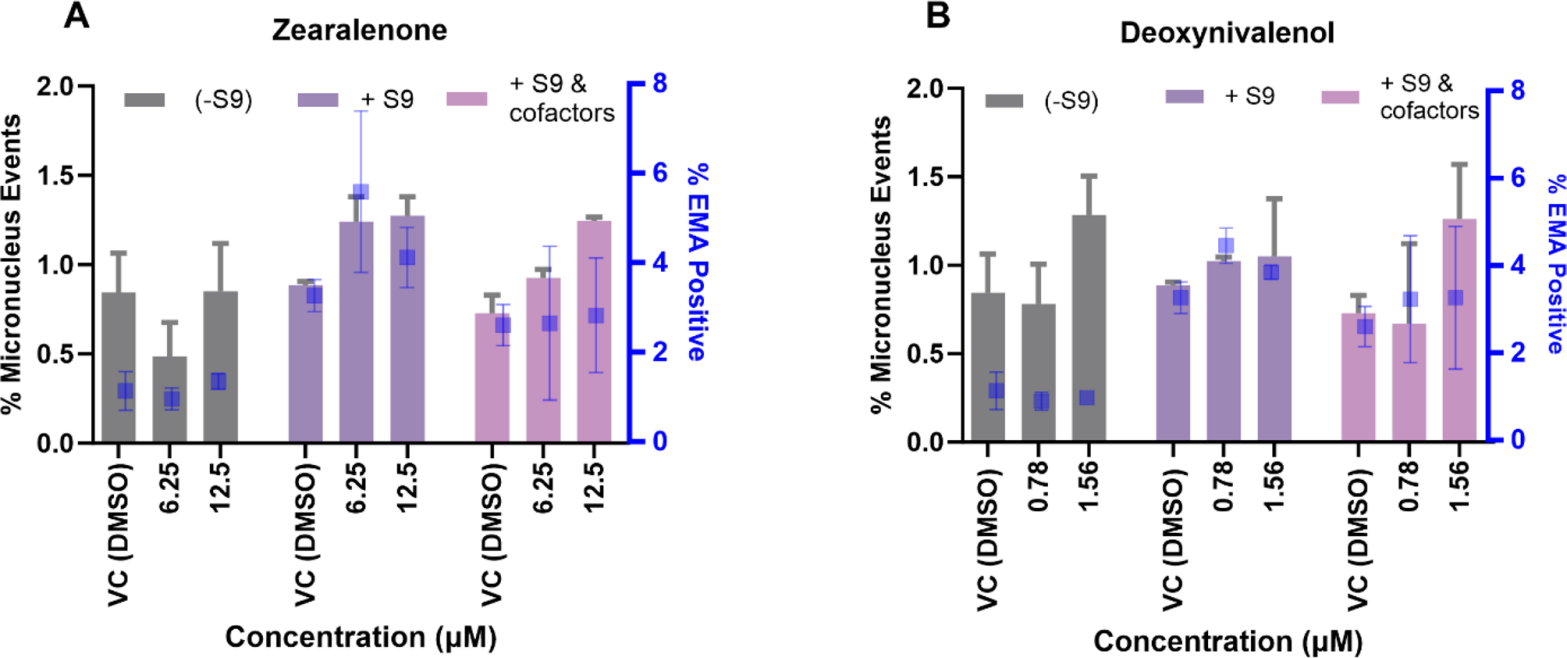
Micronuclei formations (left y-axis) and cytotoxicity (right y-axis) in TK6 cells following short-term exposure (5 h) to ZEN (**A**), and DON (**B**) in the absence of MAS (dark grey bars), in the presence of S9 only (dark purple bars), and in the presence of S9 with cofactors (light purple bars). For micronuclei formations, data bars for each test concentration presented as mean ± SD (*n* = 3). Within each treatment group, the means of each test concentration were compared to the mean vehicle control of that treatment group via pair-wise comparisons. For cytotoxicity, the scoring criteria was set as 4-fold increase in %EMA-Positive over the vehicle control of each treatment group. Blue square symbols of the %EMA-Positive results for each test concentration are presented as mean ± SD. Note that the blue square symbols have been intentionally off-set from the center of each data bars to minimize overlap of the respective SD bars

The genotoxic potentials of ZEN and DON were further evaluated in the presence of exogenous MAS for select concentrations: 6.25 and 12.50 µM for ZEN, and 0.78 and 1.56 µM for DON. In the MAS experiments, a shorter incubation period of 5 h was used, in accordance with the recommendations of the OECD TG 487. The results are shown in Fig. 6. In these experiments, neither ZEN nor DON alone or with the S9 alone or with cofactors had any statistically significant effect on % MN compared to the vehicle controls.

## Discussion

### Estrogenicity of the ZEN compounds and effect of exogenous metabolic activating systems

To better understand the effects of exogenous MAS on the endocrine activities of the ZEN compounds, a brief overview regarding the general metabolism of ZEN is provided herein. ZEN undergoes extensive phase I (e.g., reduction, oxidation) biotransformation in the liver (EFSA 2017a) including reduction of the keto group in position 7 and the double bond in position 11 and 12 by 3α- and 3β-hydroxysteroid dehydrogenases (3α- and 3β-HSD) (Kiessling and Pettersson 1978; Malekinejad et al. 2005) to form a variety of metabolites including α-ZEL and other minor forms (Han et al. 2022). These phase I metabolites, which have higher or lower estrogenic activities than ZEN, undergo further phase II metabolism to be conjugated to glucuronic acid or sulfate (EFSA 2011; Han et al. 2022). Phase II metabolism is considered to be a key mechanism in the detoxification of ZEN (Frizzell et al. 2015). ZEN can also be monohydroxylated in the aromatic position by cytochrome P450s (CYPs). Both human and rat liver microsomes can convert ZEN in vitro to 13-OH-ZEN and 15-OH-ZEN, which are less estrogenic than ZEN (Drzymala et al. 2015; Metzler et al. 2010; Pfeiffer et al. 2007; Yang et al. 2017).

#### ER agonism

In the current study, strong estrogenic activities in the picomolar range were detected in both of the ZEN compounds, with α-ZEL being the most estrogenic and almost 10 times more potent than ZEN. These results are expected as both compounds have well-documented estrogenic properties with α-ZEL being the most potent both *in vitro* and *in vivo* (Demaegdt et al. 2016; EFSA 2011; Frizzell et al. 2011; Molina-Molina et al. 2014; Shier et al. 2001; Takemura et al. 2007). From the perspective of presence in a water sample, the EC_10_ value range of 30–40 pM of ZEN detected by this bioassay converts to a concentration range of 10–12 ng/L, or approximately 0.2 ng/L if the water sample was to be enriched 50 times, which is a usual practice in our laboratory (Oskarsson et al. 2021). Levels of ZEN in environmental water samples have been reported to range from 0.5 to 80.6 ng/L (Bucheli et al. 2008; Gromadzka et al. 2009; Maragos 2012; Waśkiewicz et al. 2012). Elsewhere, drinking water inoculated with *Fusarium graminearum* was reported to synthesize 15 ng/L of ZEN (Russell and Paterson 2007). Thus, the ER bioassay is an extremely sensitive method to detect the presence of ZEN compounds in water.

The highest decrease in the estrogenicity of ZEN was observed in the presence of S9 and both PHI and II cofactors (Table 2). This is as expected given that ZEN undergoes both phase I and II biotransformation reactions which results in conjugation and detoxification (EFSA 2011, 2016). In another *in vitro* study using the MMV-Luc and TM-Luc reporter gene cell lines, a loss of estrogenic activities was reported in the glucuronide conjugates of ZEN (Frizzell et al. 2015). It should be mentioned that a decrease in the estrogenic activity of ZEN was observed at higher concentrations after incubation with S9 only in the current study, indicating a partial binding to S9 proteins. However, the remaining non-bound ZEN compounds would have still been available for metabolism. When comparing also the 95% CIs of the EC_10_ values of ZEN in the absence of MAS and in the presence of the S9 alone, there was an overlap (Table 2).

The presence of S9 with PHI cofactors alone also reduced the estrogenic activities of ZEN. This is a rather striking finding as α-ZEL is one of the expected primary phase I metabolites of ZEN and is known to be more estrogenic than ZEN. This finding may be therefore due to several reasons. For one, many different metabolites are formed following the phase I biotransformation of ZEN, (EFSA 2011; Li et al. 2020) including other novel metabolites (Pfeiffer et al. 2007). Hydroxylation, in addition to reduction, has been identified as one of the main metabolic pathways of ZEN in vitro and results in several hydroxylated metabolites such as the monohydroxylated ZEN, 8-OH-ZEN, 13-OH-ZEN, and 15-OH-ZEN (Hildebrand et al. 2012; Pfeiffer et al. 2007, 2009; Yang et al. 2017). As previously mentioned, these metabolites are less estrogenic than ZEN. The estrogenic potency of 15-OH-ZEN, for instance, has been reported to be 0.02 compared to ZEN, set at 1 (Drzymala et al. 2015). In another study, the *in vitro* formation of OH-ZEN was reported to be highest in phenobarbital (PB)-treated rat liver microsomes (Bravin et al. 2009), which were used also in our study. Interestingly, in that same preparation, α-ZEL was not detected. Another important consideration is the enzymatic reactions involved in the hepatic biotransformation of ZEN. As previously mentioned, 3α- and 3β-HSD have been identified as key enzymes in the reduction of ZEN, and these steroid hormone reductase activities can differ depending on the species source of S9, the cofactor, as well as the incubation pH (Malekinejad et al. 2005, 2006; Ueno et al. 1983). As such, it may be that the experimental conditions in our study did not favor the production of α-ZEL. Further to this, differences in the prevailing metabolites as well as amounts of the metabolites formed from ZEN have been reported between animal species, as summarized in EFSA (2011, 2017a), Rai et al. (2020), and Yang et al. (2017). Greater amounts of α-ZEL are reported to form in humans as well as in pigs and other domestic animal species compared to in rodents (Kuiper-Goodman et al. 1987; Malekinejad et al. 2006). This is relevant to our study as we utilized S9 fractions derived from rats. Finally, another factor may be due to the assay method in the current study in that the cells were continuously exposed (24 h) to the test compound with the MAS, which may not reflect the duration of the actual biotransformation process that occurs *in vivo*. In the presence of NADPH, ZEN has been shown to be rapidly metabolized by rat liver microsomes based on depletion kinetics with a decrease of substrate concentration to approximately 40% after only 60 min (Drzymala et al. 2014).

In the current study, similar to what was observed with ZEN, the estrogenicity of α-ZEL was reduced most in the presence of S9 and both PHI and II cofactors. Elsewhere, spectral analysis of the metabolic behavior of α-ZEL identified glucuronidation as the prevailing detoxification pathway (Yang et al. 2020). This is in general agreement with our findings that the estrogenicity of α-ZEL was most reduced in the presence of the PHII cofactors. 

#### ER antagonism

Anti-estrogenic activities were detected for both ZEN compounds in the current study, but at much higher concentrations than what was found for the estrogenic effects. This observation is similar to what was reported by Demaegdt et al. (2016). Here, the order of relative potencies for anti-estrogenic activities was also consistent to that observed for estrogenicity with α-ZEL being more potent than ZEN. Further, similar to what was observed for ER agonism in the presence of MAS, the ER antagonistic effects of ZEN and α-ZEL were reduced most in the presence of S9 with the PHI and II cofactors and also in the presence of S9 with the PHI cofactors alone. The fact that these observed patterns are consistent between the agonist and antagonist modes validates that the incorporation of the exogenous MAS was successfully implemented. The anti-estrogenic activities of the two ZEN compounds as well as following metabolism occurred at several fold higher concentrations in comparison to their estrogenicity and are therefore not of the same health concern. Regardless, ZEN has been reported to be a mixed agonist/antagonist of the estrogen receptor (Mueller et al. 2004), and specifically antagonistic for the ERβ subtype in transactivation assays (Kuiper et al. 1998). In fact, no antagonistic activity of ZEN could be detected when HEK-293 cells were transfected with the ERα subtype (Kuiper et al. 1998). The VM7Luc4E2 cell line used in the current study expresses both ERα and ERβ subtypes (Brennan et al. 2016).

### Androgenicity of the ZEN compounds and effect of exogenous metabolic activating systems

While no AR agonism without and with MAS was observed, AR antagonism (in the µM range) was detected from both ZEN compounds with α-ZEL being more potent than ZEN. In the presence of the MAS, the anti-androgenic effects of both compounds followed the same pattern as reported in the ER assay in that the activities were most decreased in the presence of the S9 with PHI and II cofactors, and to a lesser extent by S9 with the PHI cofactors alone. Elsewhere, ZEN and α-ZEL were reported to exhibit a weak antagonistic effect on the AR in the TARM-Luc cell line (Frizzell et al. 2011) as well as in the androgen-sensitive PALM cells with IC_50_ values in the micromolar range (Molina-Molina et al. 2014). In that 2014 same study, no androgenic activities were detected from ZEN (or any of its five metabolites) in the PALM cells after 40 h of exposure at concentrations up to 10 µM. The literature regarding the androgenicity of ZEN and α-ZEL (and other metabolites) is otherwise lacking. Thus, the findings in the current study on the similar effects of MAS in both the ER and AR assays are interesting and suggest that both the phase I and phase II pathways are important in the detoxification of the estrogenicity as well as androgenicity of the ZEN compounds.

### Estrogenicity of the DON compounds and effect of exogenous metabolic activating systems

As with the discussion regarding the ZEN compounds, background information regarding the metabolism of DON is provided briefly herein. To begin, 3-Ac-DON and 15-Ac-DON are primarily deacetylated to DON in the intestines prior to distribution and are thus expected to have the same acute and chronic effects as DON (EFSA 2017b). DON itself is metabolized in the liver mainly by phase II metabolism with de-epoxidation, glucuronide, and sulfonate conjugation considered key metabolic pathways (Yao and Long 2020). The microsomal formation of DON glucuronides in vitro has been reported in rats, as well as other animal species and humans (Maul et al. 2012, 2015).

#### ER agonism and antagonism

For 3-Ac-DON, estrogenicity was detected (albeit nearly 10^5^ times lower than α-ZEL and only 30% of the maximum response of E2 in the assay), and this activity was deactivated by S9 with PHI and II cofactors as well as by S9 with PHII cofactors alone. While there is limited literature on the metabolism and toxicity of the acetylated and modified forms of DON, it has been observed that in rats, *in vivo* hydrolyzation of the acetylated forms in the stomach releases DON and also that the acetylated forms of DON can be directly glucuronidated (i.e., phase II biotransformation) to form modified metabolites (Veršilovskis et al. 2012). In the current study, no ER agonistic effects of DON and 15-Ac-DON were observed and the incorporation of MAS had no effect either. The fact that DON exhibited no estrogenic activities in our study is in general agreement with a few other recent studies using similar reporter gene assays (Demaegdt et al. 2016; Ndossi et al. 2012). Anti-estrogenic effects from DON and 15-Ac-DON were detected in the current study. Somewhat strikingly, increased antagonistic effects were observed for both compounds in the presence of S9 with PHII cofactors alone. While the hormonal effects of the DON compounds using *in vitro* reporter gene assays have not been readily studied elsewhere to compare our results to, limited other studies related to reproductive toxicity have shown that DON impairs steroidogenesis in vitro in porcine ovarian granulosa cells (Kolesarova et al. 2017) and induces toxic effects *ex vivo* in the follicular development of porcine ovaries (Gerez et al. 2017).

### Androgenicity of the DON compounds and effect of exogenous metabolic activating systems

No AR antagonistic activities were detected in any of the DON compounds, while AR agonistic activities were seen (in the micromolar range). The agonistic effects of the DON compounds increased slightly in the presence of S9 with the PHII cofactors alone as well as in the presence of S9 with the PHI and II cofactors. While there is a lack of other *in vitro* reporter gene studies to compare our results to, the potential of DON to act as an endocrine disruptor has been linked to its effects on steroidogenesis (Ndossi et al. 2012). *In vivo*, DON has been reported to adversely affect various endpoints of male reproductive function in the rat model, although the mechanisms leading to the effects were not known (Sprando et al. 2005).

### Genotoxicity assessments of ZEN and DON

#### ZEN

In the current study, genotoxicity was observed for one of the test concentrations (12.5 µM) of ZEN in the MN assay without MAS after a 24-h exposure period. The genotoxic potential of ZEN has been investigated in various other *in vitro* assays (Rencüzoğulları and Aydin 2019) and has been determined to be clastogenic by EFSA’s Panel on Contaminants in the Food Chain (EFSA 2011). However, the potential of ZEN to cause micronucleus formations remains inconclusive (Rencüzoğulları and Aydin 2019; Ülger et al. 2020). In one study involving Vero monkey kidney cells, a dose-dependent induction of binucleated micronucleated cells by ZEN was observed in the cytokinesis block micronucleus assay (Ouanes et al. 2003). In that study, the cells were exposed to 5, 10, and 20 µM of ZEN. However, no information regarding the cytotoxicity of the test concentrations was provided to compare our results to. Elsewhere, ZEN concentrations of 10, 20, and 40 µM were demonstrated to induce cell cycle arrest in three different mammalian cell lines (Abid-Essefi et al. 2003).

In addition to assessing the genotoxic potential of ZEN, the impact of exogenous MAS was also tested. However, the exogenous MAS had no statistically significant effect on the % MN, which would suggest that the metabolites of ZEN formed in the present study are not genotoxic. It is noteworthy to mention that no genotoxicity was observed at the 12.5-µM test concentration of ZEN in the experiments incorporating MAS, which is inconsistent with what was observed in the initial trials without MAS, as described earlier in this section. This discrepancy is probably attributed to the differences in exposure periods. In the initial trials without MAS, the cells were continuously exposed to the test compound for a period of 24 h. However, in the trials incorporating the exogenous MAS, the cells were exposed to the test compound for a shorter period (5 h).

### DON

For DON, no genotoxic effects were detected at any of the non-cytotoxic test concentrations in the initial trials or in the presence of the exogenous MAS in the current study. In another study that also utilized the MN assay following the OECD 487 and in the same cell line (TK6), DON did not induce any significant MN formations at concentrations below 12.5 µM after 3 h of treatment either with or without human and rat liver S9 (Takakura et al. 2014). The fact that the exogenous MAS had no effect in our study correlates well with this conclusion. Cytotoxicity was also observed in that study, which is consistent with what was observed within the same concentration range in our study. It should be mentioned that DON can otherwise bind to ribosomes, thereby inhibiting protein synthesis and subsequent RNA and DNA synthesis (EFSA 2017b). The genotoxic potential of DON has been investigated in numerous other *in vitro* assays, and the EFSA Panel on Contaminants in the Food Chain considers it to be genotoxic *in vitro*, while the available data on its genotoxicity *in vivo* remains inconclusive (EFSA 2017b).

In summary, the effects of ZEN, DON, and their primary derivatives on the estrogen and androgen receptors and induction of micronuclei in the presence of exogenous MAS were investigated. Here we demonstrated a reduction of estrogenic and anti-estrogenic effects of ZEN and α-ZEL following the phase I reaction, which was further reduced after the phase II reaction. The decrease in the estrogenic effects of ZEN following the phase I reaction may be explained by the production of mono-hydroxylated metabolites with low estrogenic activities. In terms of genotoxicity, the current study did not find any induction of micronuclei formations from ZEN or DON in the presence of exogenous MAS. It may be worthwhile to mention that the PHI and II cofactor solutions incorporated into the hormonal receptor assays differed from that used in the MN assay (e.g., not sourced from the same suppliers). Overall, our study highlighted that the inclusion of a metabolic activation system is a useful tool to assess biological effects of metabolites in *in vitro* bioassays.

It should also be highlighted that the *in vitro* ER bioassay used in the current study proved to be a highly sensitive method to detect low concentrations (in the pM range) of the ZEN compounds in aqueous solutions. This and the other *in vitro* assays used in our study are commonly applied as effect-based methods in water quality monitoring and assessments (GWRC 2020a). The approach involves the collection of water samples which are often concentrated by some form of phase extraction (e.g., solid- or liquid-phase extraction techniques) and then completed with a solvent such as DMSO (GWRC 2020b). This is useful as mycotoxins in water bodies have been identified as emerging contaminants to be given more attention, particularly with respect to drinking water sources (Mhlongo et al. 2019; Székács 2021). As such, future direction with this project can involve the application of these *in vitro* assays to investigate the same endocrine endpoints from mycotoxins present in surface and drinking water samples, especially the estrogenic activity of ZEN compounds.

### Supplementary Information

Below is the link to the electronic supplementary material.

Supplementary file1 (DOCX 862 KB)
