# Correction: Estrogenic, androgenic, and genotoxic activities of zearalenone and deoxynivalenol in in vitro bioassays including exogenous metabolic activation

**DOI:** 10.1007/s12550-024-00535-4

**Published:** 2024-04-26

**Authors:** Maria Yu, Agneta Oskarsson, Jan Alexander, Johan Lundqvist

**Affiliations:** 1 Department of Biomedical Sciences and Veterinary Public Health, Swedish University of Agricultural Sciences, Box 7028, SE-750 07 Uppsala, Sweden; 2 Norwegian Scientific Committee for Food and Environment, Norwegian Institute of Public Health, P.O. Box 222 Skøyen, NO-0213 Oslo, Norway

**Correction to: Mycotoxin Research** 10.1007/s12550-024-00529-2

In Tables 2 and 3 of this article, the data “LOD” are missing in multiple occurrences. Given below are the corrected tables.

**Table 2:** Summary of effect concentrations (EC_10_ for receptor agonism and IC_30_ for receptor antagonism) of the ZEN compounds in the ER and AR assays without and in the presence of an exogenous MAS. 95% confidence intervals [lower limit or LL, upper limit or UL] of the EC_10_/IC_30_ values are presented in brackets below each value

**Test Compound**	**MAS**	**ER agonism** **EC**_**10**_** (pM)**				**ER antagonism** **IC**_**30**_** (µM)**				**AR agonism** **EC**_**10**_** (pM)**				**AR antagonism** **IC**_**30**_** (µM)**			
	**S9**	**-**	**+**	**+**	**+**	**-**	**+**	**+**	**+**	**-**	**+**	**+**	**+**	**-**	**+**	**+**	**+**
	**+ PHI**	**-**	**-**	**+**	**+**	**-**	**-**	**+**	**+**	**-**	**-**	**+**	**+**	**-**	**-**	**+**	**+**
	**+ PHII**	**-**	**-**	**-**	**+**	**-**	**-**	**-**	**+**	**-**	**-**	**-**	**+**	**-**	**-**	**-**	**+**
**ZEN**		46.5 [29.5,72.4]	63.3 [56.2,97.2]	984 [740,1 279]	6 472 [4 121,9 506]	2.92 [2.09,3.89]	3.41 [2.40,4.68]	10.3 [8.71,11.8]	16.2 [6.76,16.2]	< LOD	< LOD	< LOD	< LOD	2.37 [1.95,2.75]	2.14 [1.82,2.45]	7.33 [6.31,8.13]	18.0 [13.8,23.4]
**α-ZEL**		3.70 [2.51,5.37]	4.24 [2.82,6.31]	49.3 [30.2,75.9]	155.1 [93.3,245.4]	1.03 [0.33,1.78]	1.75 [0.69,3.02]	2.42 [1.35,3.63]	5.10 [2.88,8.13]	< LOD	< LOD	< LOD	< LOD	0.95 [0.85,1.05]	0.74 [0.62,0.87]	1.12 [0.91,1.32]	1.61 [1.10,2.14]

**Table 3:** Summary of effect concentrations (EC_10_ for receptor agonism and IC_30_ for receptor antagonism) of the DON compounds in the ER and AR assays without and in the presence of exogenous MAS. 95% confidence intervals [LL, UL] of the EC_10_/IC_30_ values are presented in brackets below each value

**Test Compound**	**MAS**	**ER agonism** **EC**_**10**_** (µM)**				**ER antagonism** **IC**_**30**_** (µM)**				**AR agonism** **EC**_**10**_** (µM)**				**AR antagonism** **IC**_**30**_** (µM)**			
	**S9**	**-**	**+**	**+**	**+**	**-**	**+**	**+**	**+**	**-**	**+**	**+**	**+**	**-**	**+**	**+**	**+**
	**+ PHI**	**-**	**-**	**+**	**-**	**-**	**-**	**+**	**-**	**-**	**-**	**+**	**-**	**-**	**-**	**+**	**-**
	**+ PHII**	**-**	**-**	**+**	**+**	**-**	**-**	**+**	**+**	**-**	**-**	**+**	**+**	**-**	**-**	**+**	**+**
**DON**		< LOD	< LOD	< LOD	< LOD	0.81 [0.69,1.00]	0.75 [0.54,1.05]	0.37 [0.25,0.58]	0.25 [0.18,0.38]	0.87 [0.81,0.93]	0.84 [0.72,0.98]	0.45 [0.39,0.52]	0.38 [0.32,0.46]	< LOD	< LOD	< LOD	< LOD
**3-Ac-** **DON**		0.45 [0.41,0.49]	0.53 [0.49,0.59]	< LOD	< LOD	< LOD	< LOD	< LOD	< LOD	1.11 [0.95,1.26]	1.07 [0.91,1.23]	0.91 [0.65,1.35]	0.63 [0.52,0.78]	< LOD	< LOD	< LOD	< LOD
**15-Ac-DON**		< LOD	< LOD	< LOD	< LOD	1.09 [0.98, 1.20]	1.11 [0.81,1.38]	0.48 [0.30,0.72]	0.24 [0.18,0.32]	0.81 [0.74,0.87]	0.70 [0.62,0.76]	0.52 [0.45,0.62]	0.50 [0.45,0.56]	< LOD	< LOD	< LOD	< LOD

The original article has been corrected.

