# Supplementary material for: Estrogenic, androgenic, and genotoxic activities of zearalenone and deoxynivalenol in in vitro bioassays including exogenous metabolic activation
Source: Mycotoxin Res. 2024 Apr 8;40(3):331–46. doi: 10.1007/s12550-024-00529-2 (PMC11258189; doi:10.1007/s12550-024-00529-2)
Supplement: Supplementary file 1 — Supplementary file1 (DOCX 862 KB) [file 12550_2024_529_MOESM1_ESM.docx]

**Estrogenic, androgenic and genotoxic activities of zearalenone and deoxynivalenol in *in vitro* bioassays including exogenous metabolic activation**

**Supplementary Information**

**Maria Yu^a*^, Agneta Oskarsson^a^, Jan Alexander^b^, and Johan Lundqvist^a^**

^a^ Department of Biomedical Sciences and Veterinary Public Health, Swedish University of Agricultural Sciences, Box 7028, SE-750 07 Uppsala, Sweden

^b^ Norwegian Scientific Committee for Food and Environment, Norwegian Institute of Public Health, P.O. Box 222 Skøyen, NO-0213 Oslo, Norway

*Corresponding author:

Maria Yu

Department of Biomedical Sciences and Veterinary Public Health, Swedish University of Agricultural Sciences, Box 7028, SE-750 07 Uppsala, Sweden.

E-mail address: [maria.yu@slu.se](mailto:maria.yu@slu.se)

1. **Detailed information on cell lines and bioanalyses**
   1. ***Cell lines and maintenance***

The VM7Luc4E2 cell line, derived from the MCF7 human breast cancer cell line, was kindly donated by the late M. Denison (University of California, CA, USA) and was used in the estrogenic receptor (ER) assay. The cells were cultured as monolayers in Roswell Park Memorial Institute (RPMI) 1640 medium with L-glutamine (Thermo Fisher Scientific, Waltham, USA) supplemented with 8% FBS (Thermo Fisher Scientific, Waltham, USA), 0.9% penicillin-streptomycin (5,000 units/mL penicillin, 5,000 µg/mL streptomycin) (Thermo Fisher Scientific, Waltham, USA), and 0.55 mg/mL Gentamicin (Gentamicin Sulfate, 50 mg/mL, Thermo Fisher Scientific, Waltham, USA)) as a positive selector. Two to three days before conducting the experiments, cells were transferred to experimental medium consisting of Dulbecco’s Modified Eagle’s Medium (DMEM) with 4.5 g/L glucose and no glutamine or phenol red (Thermo Fisher Scientific, Waltham, USA) supplemented with 4.5% dextran-charcoal treated FBS (Thermo Fisher Scientific, Waltham, USA)), 1.9% L-glutamine (Thermo Fisher Scientific, Waltham, USA), 0.9% penicillin-streptomycin (5,000 units/mL penicillin, 5,000 µg/mL streptomycin), and 0.38 mg/mL Gentamicin.

The AR EcoScreen GR-KO M1 cell line (JCRB1761), which are stably transfected Chinese hamster ovary (CHO) cells with GR knockout gene, was used in the androgen receptor (AR) assay. The cells were obtained from the JCRB (JCRB1761). Cells were maintained as monolayers in Dulbecco's Modified Eagle Medium: Nutrient Mixture (DMEM) F-12 medium (Sigma-Aldrich, Darmstadt, Germany) supplemented with 10% FBS, 2% penicillin-streptomycin (5,000 units/mL penicillin, 5,000 µg/mL streptomycin), 1% L-glutamine, 50 µg/mL Zeocin (InvivoGen, Toulouse, France) and 25 µg/mL Hygromycin B Gold (InvivoGen, Toulouse, France). The experimental medium contained DMEM-F12 (Sigma-Aldrich, Darmstadt, Germany), 10% dextran-charcoal treated FBS, 1% penicillin-streptomycin and 1% L-glutamine.

The human lymphoblast thymidine kinase (TK6) heterozygote cell line (American Type Culture Collection, ATCC, Manassas, USA) was used in the micronucleus assay. The cells were cultured in suspension in medium consisting of RPMI-1640 medium with L-glutamine (Thermo Fisher Scientific, Waltham, USA)) supplemented with 10% FBS and 1% penicillin-streptomycin (5,000 units/mL penicillin, 5,000 µg/mL streptomycin). The cell cycle was monitored daily and the doubling time was calculated to be approximately 10-12 h. The experimental medium contained the same components as the culture medium.

For cells maintained as monolayers, medium was replaced every 2-3 days and cells were sub-cultured twice a week. Once thawed, all cell lines were maintained up to a maximum of thirty passages. Cells grown in suspension were maintained at a density at or below 10^6^ cells/mL and medium was renewed daily. All cells were grown/incubated in a humidified atmosphere at 37 °C and 5% CO_2_.

- 1. **Description of bioassays**
     1. ***Reporter gene assays for endocrine effects***

The estrogen and androgen receptor effects of the test compounds were measured using luciferase-based reporter gene assays on the stably transfected cell lines. All experiments, without and with the exogenous metabolic components, were conducted over a 3-day period. On day 1, cells were trypsinized and incubated in experimental medium for 24 h. VM7Luc4E2 cells were plated at a density of 16,000 cells/well and 4,000 cells/well for the AR EcoScreen GR KO M1 cells, both in white 384-well plates (Costar® Corning Incorporated, Kennebunk, USA). On day 2, cells were treated with the test compounds and reference compounds then incubated for another 24 hrs. At experiment termination on day 3, cells were lysed with 10 µL/well with passive lysis buffer (PLB) (Promega, Nacka, Sweden) and the plates were shaken for 15-30 minutes. Luciferase activity was measured using the Luciferase® Reporter Assay System (Promega) according to the manufacturer’s instructions. Luminescence was measured on the TECAN Spark® Multimode Microplate Reader (TECAN, Austria, GmbH, SparkControl version 3.1 software) with an automatic injection syringe. The injection volume for the Firefly luciferase reagent was 10 µL/well for 384-well plates. Luminescence measurement was conducted over a 5 s period, 2 s after the reader was automatically injected with Firefly luciferase reagent. White adhesive sealing film was attached to plate bottoms before readings were taken.

In all experiments, vehicle controls consisting of 1% DMSO, equivalent to the DMSO concentration in the test compound solutions and reference compounds were included. All test compounds, vehicle controls, and reference compounds were tested in quadruplicates/plate.

- - 1. ***Cell viability assays***

Cytotoxicity of the test compounds in the VM7Luc4E2 and AR-EcoScreen GR KO M1 cell lines was tested by the ATP-based assay (CellTiter-Glo® Luminescent Cell Viability Assay, Promega, Nacka, Sweden). The plating densities for each cell line and 3-day treatment schedule were the same as described above and all were plated in white 384-well plates. Following exposure, 25 µL of CellTiter-Glo® Luminescent Cell Viability Assay (Promega, Nacka, Sweden) was added to each well and the plates were shaken for 15 to 20 min then left to stand for 2 min thereafter. A white adhesive sealing film was attached at the bottom of the 384-well plate, then luminescence was measured on the TECAN Spark® Multimode Microplate Reader (TECAN, Austria, GmbH, SparkControl version 3.1 software).

- - 1. ***Micronucleus assay for genotoxicity***

The genotoxic potentials of the test compounds (ZEN and DON) were assessed using the micronucleus assay and flow cytometry in the human lymphoblast TK6 cells. The experiments, without and with the exogenous metabolic components, were conducted over a 2-day period. On day 1, cells were plated at a density of 20,000 cells/well in transparent round-bottomed 96-well plates (Sarstedt, Nümbrecht, Germany) and immediately exposed to the test compounds in the absence of the exogenous metabolic components for 24 h in the experiments without the metabolic components. For the experiments wherein the exogenous metabolic components were incorporated, the cells were plated and incubated for approximately 8 h then exposed to the test compounds and metabolic components for approximately 5 h. This was done to ensure that the cells were exposed to the test compounds and metabolic components during their doubling stage. At the end of the 5 h, cells were collected by centrifugation at 300 x g for 5 min. The exposure medium was then carefully aspirated from the wells and the cells rinsed with fresh experimental medium then re-collected by centrifugation. Fresh experimental medium was added to each well and the plate was incubated for the remaining 24 h. At experiment termination on day 2, cells were stained for flow cytometry analysis using the MicroFlow In Vitro Kit® (Litron Laboratories, Rochester, NY, USA) according to the manufacturer’s protocol.

In all experiments, vehicle controls consisting of 1% DMSO, equivalent to the DMSO concentration in the test compound solutions and positive controls were included. All test compounds, vehicle controls, and positive controls were tested in triplicates/plate.

Flow cytometry was performed using a FACSVerse (BD Biosciences, Franklin Lakes, NJ, USA). For each replicate, approximately 20,000 events were collected. The blue laser (488 nm) was used for excitation. Size (FSC) was collected with a diode detector while internal complexity (SSC) was collected through a 488/15 nm bandpass filter. For green fluorescence (FL1), collected through a 527/32 nm bandpass filter, area (A), height (H) and width (W) of signals were recorded, while for red fluorescence (FL3), collected through a 700/54 nm bandpass filter, the height of the signals were acquired. The time parameter was also acquired, to ensure stability of measurements. The gating strategy used was the one recommended by the manufacturer of the kit, and included gates to eliminate doublets in a FL1-W/FL1-A plot and also elimination of dead/dying cells in FL1-H/FSC, FL1-H/SSC and FL3-H/FL1-H plots. Cell cycle characteristics were monitored using a FL1-A histogram in linear scale. The final scoring of micronuclei was performed using a FL1-H/FSC plot, and expressed as percentage of micronuclei among the events meeting the gating criteria.

To assess cytotoxicity, cells were stained with ethidium monoazide (EMA) in accordance with the instructions from the kit’s manufacturer. A >4-fold EMA-positive event increase over the vehicle control, as recommended in the kit’s protocol, was applied as the cytotoxicity limit.

1. **Chemical structures**


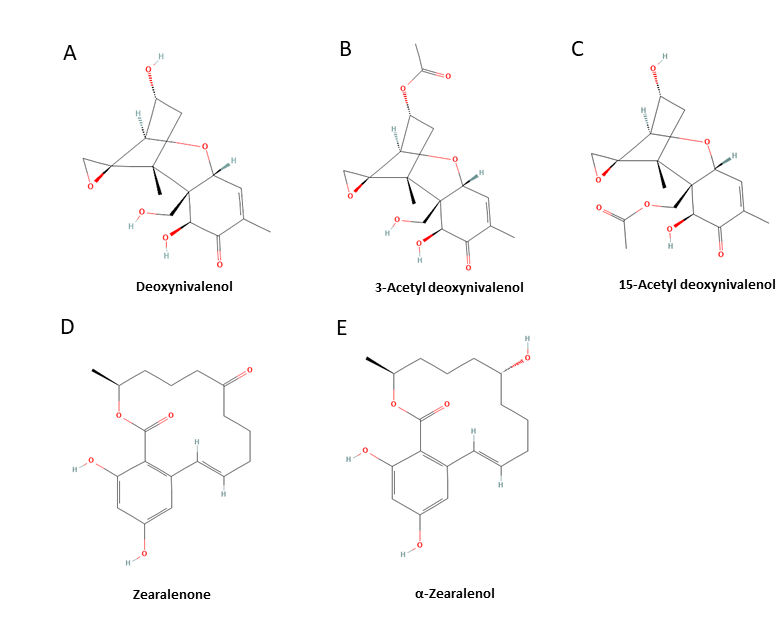


**Fig. SI-1** Chemical structures of (A) Deoxynivalenol (CAS 51481-10-8, PubChem CID 40024), (B) 3-Aceytl deoxynivalenol (CAS 50722-38-8, PubChem CID 5458510), (C) 15-Aceytl deoxynivalenol (CAS 88337-96-6, PubChem CID 10382483), (D) Zearalenone (CAS 17924-92-4, PubChem ID 5281576), and (E) α-Zearalenol (CAS 36455-72-8, PubChem ID 5284645).

1. **Cytotoxicity assessments**
   1. Initial concentration-range finding trials


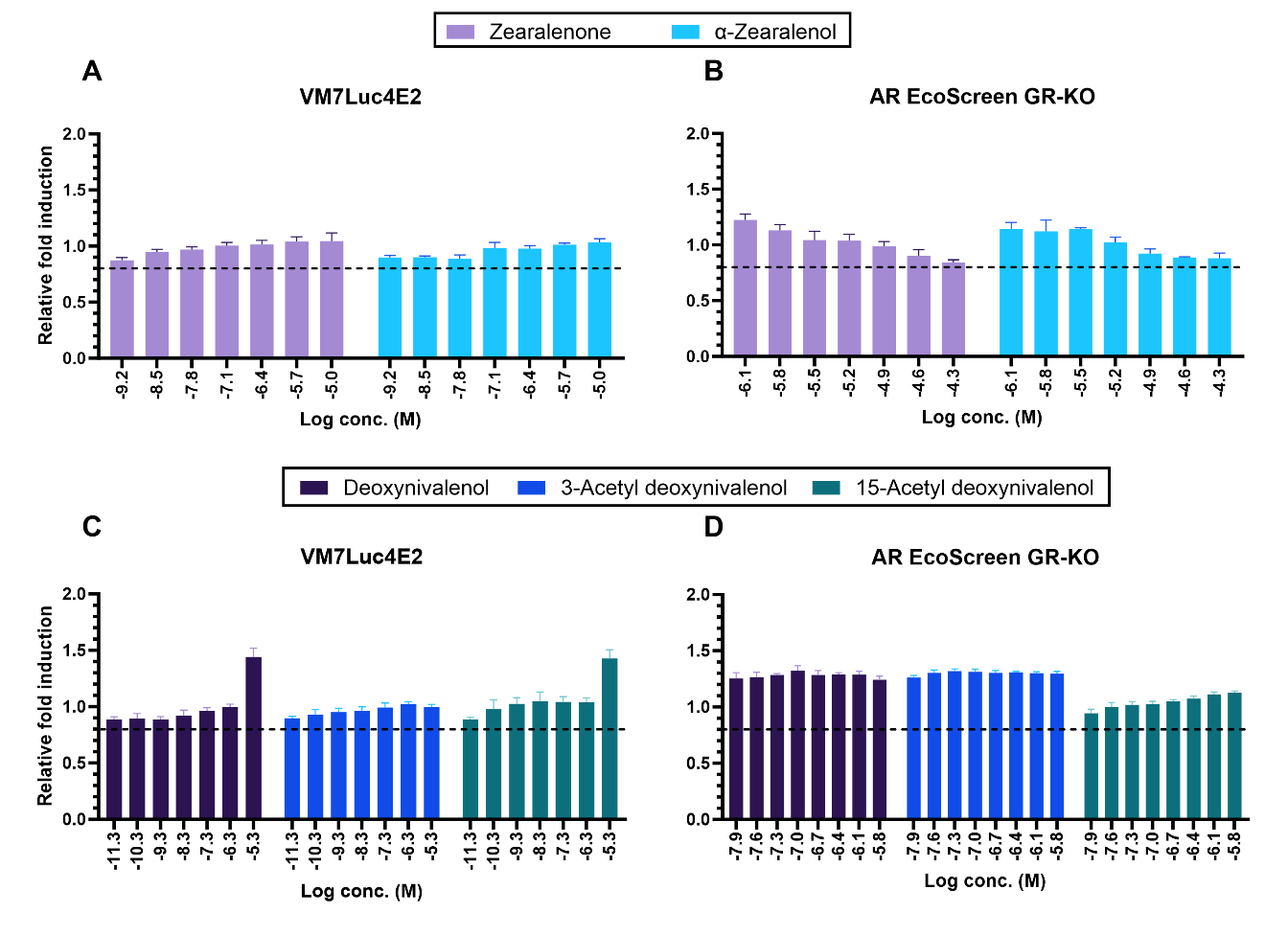


**Fig. SI-2** Cell viabilities in the VM7Luc4E2 cell line for the ZEN compounds (A) and DON compounds (C) and in the AR EcoScreen cell line for the ZEN compounds (B) and DON compounds (D). The dotted black lines indicate the cut-off values for cytotoxicity, defined as cell viability <0.80 compared to the vehicle controls. Test concentration treatments (n=4) were normalized to vehicle controls (n=8). Data presented as mean ± SD.


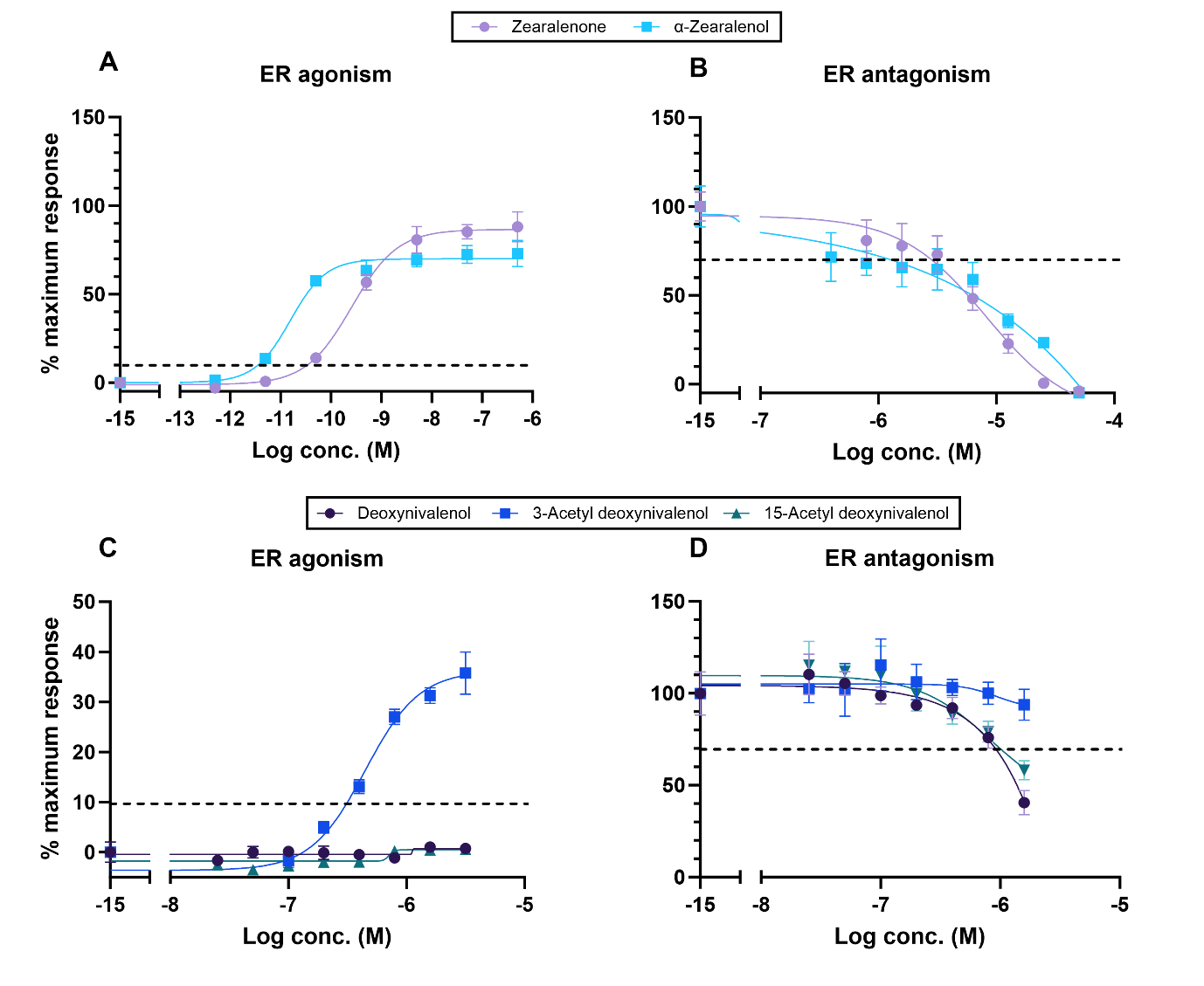


**Fig. SI-3** Concentration effect curves of the ER agonistic and antagonistic activities for the ZEN compounds (A,B) and DON compounds (C,D). Test concentration treatments (n=4) were normalized to vehicle controls (n=4). Data presented as mean ± SD.


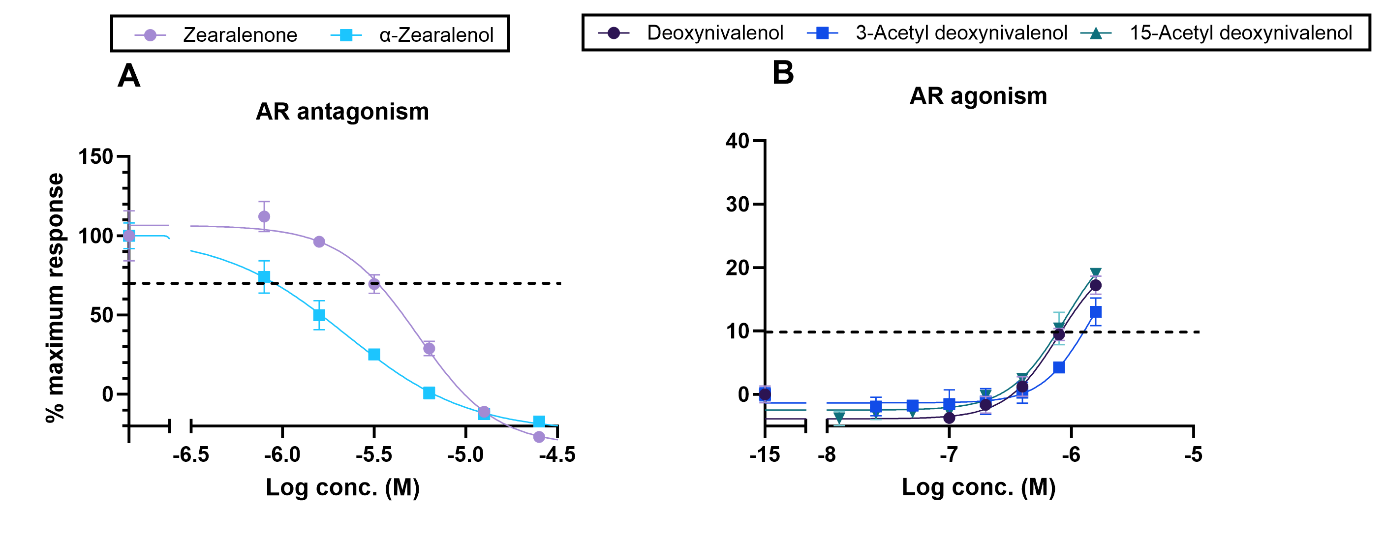


**Fig. SI-4** Concentration effect curves of the AR antagonistic activities for the ZEN compounds (A) and agonistic activities of the DON compounds (B). Test concentration treatments (n=4) were normalized to vehicle controls (n=4). Data presented as mean ± SD. No AR agonistic activities were observed for the ZEN compounds and no antagonistic activities were observed for the DON compounds, hence not graphed.

- 1. Incorporation of exogenous MAS


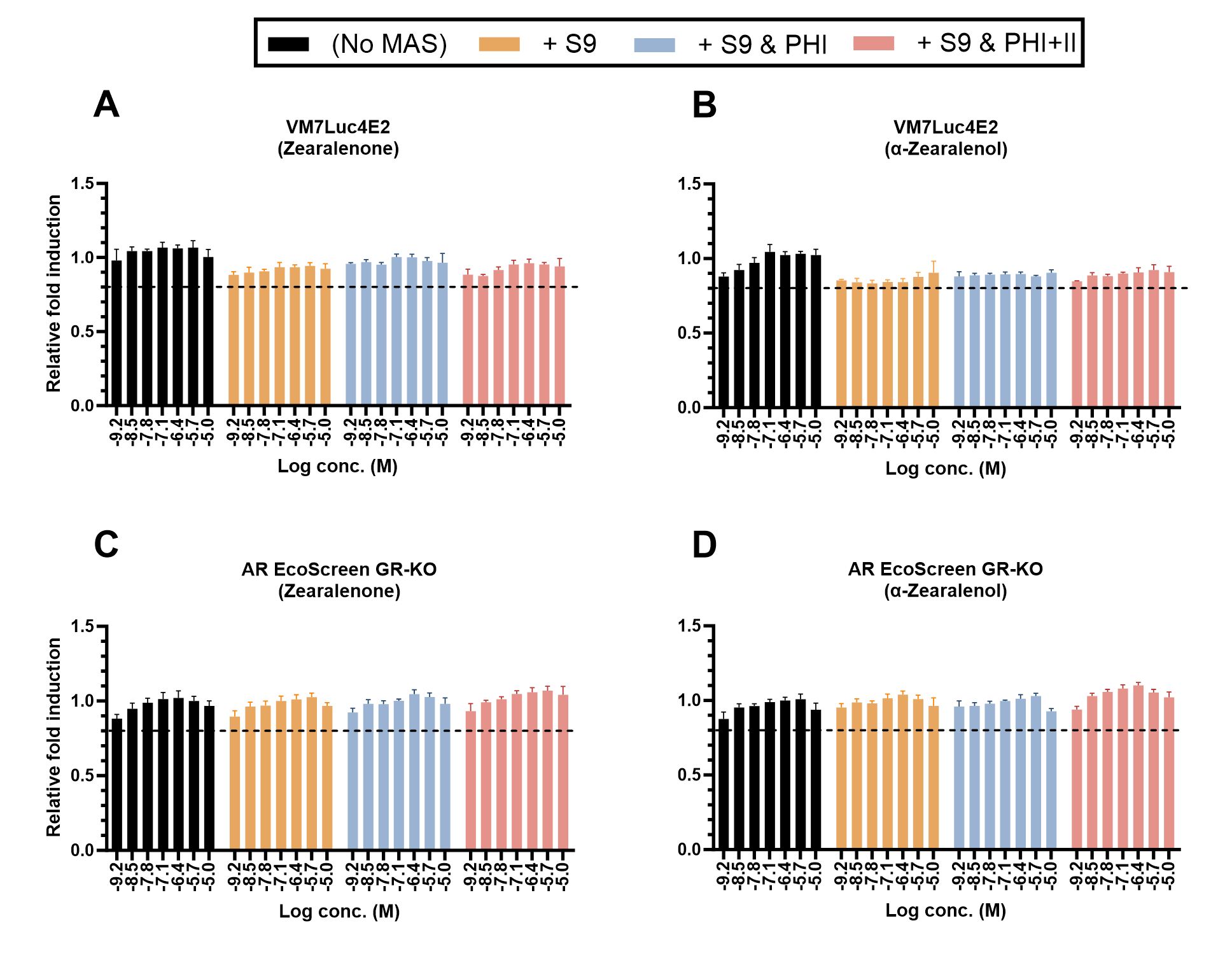


**Fig. SI-5** Cell viabilities for the ZEN compounds in the VM7Luc4E2 cell line (A,B) and AR EcoScreen cell line (C,D) without and in the presence of exogenous MAS. Each test compound was assayed in the absence of MAS (black bars), in the presence of S9 alone (light orange bars), S9 with Phase I cofactors (light blue bars), or S9 with Phase I and Phase II cofactors (light pink bars). The dotted black lines indicate the cut-off values for cytotoxicity, defined as cell viability <0.80 compared to the vehicle controls. Test concentration treatments (n=4) were normalized to vehicle controls (n=4). Data presented as mean ± SD.


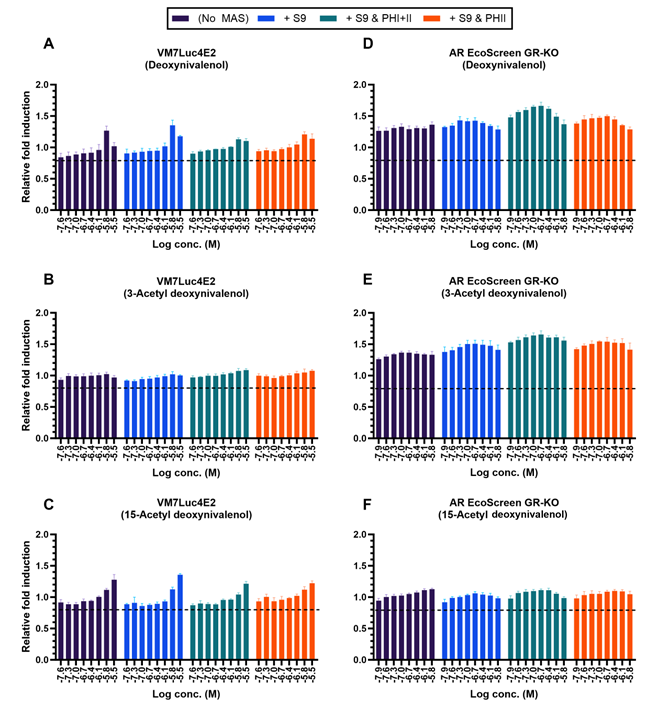


**Fig. SI-6** Cell viabilities for the DON compounds in the VM7Luc4E2 cell line (A-C) and AR EcoScreen cell line (D-F) without and in the presence of exogenous MAS. Each test compound was assayed in the absence of MAS (dark purple bars), in the presence of S9 alone (light blue bars), S9 with Phase I cofactors (teal bars), or S9 with Phase I and Phase II cofactors (orange bars). The dotted black lines indicate the cut-off values for cytotoxicity, defined as cell viability <0.80 compared to the vehicle controls. Test concentration treatments (n=4) were normalized to vehicle controls (n=4). Data presented as mean ± SD.

1. **Summary tables of effect concentrations in presence of MAS**
   1. Reference compounds with MAS for ER and AR assays


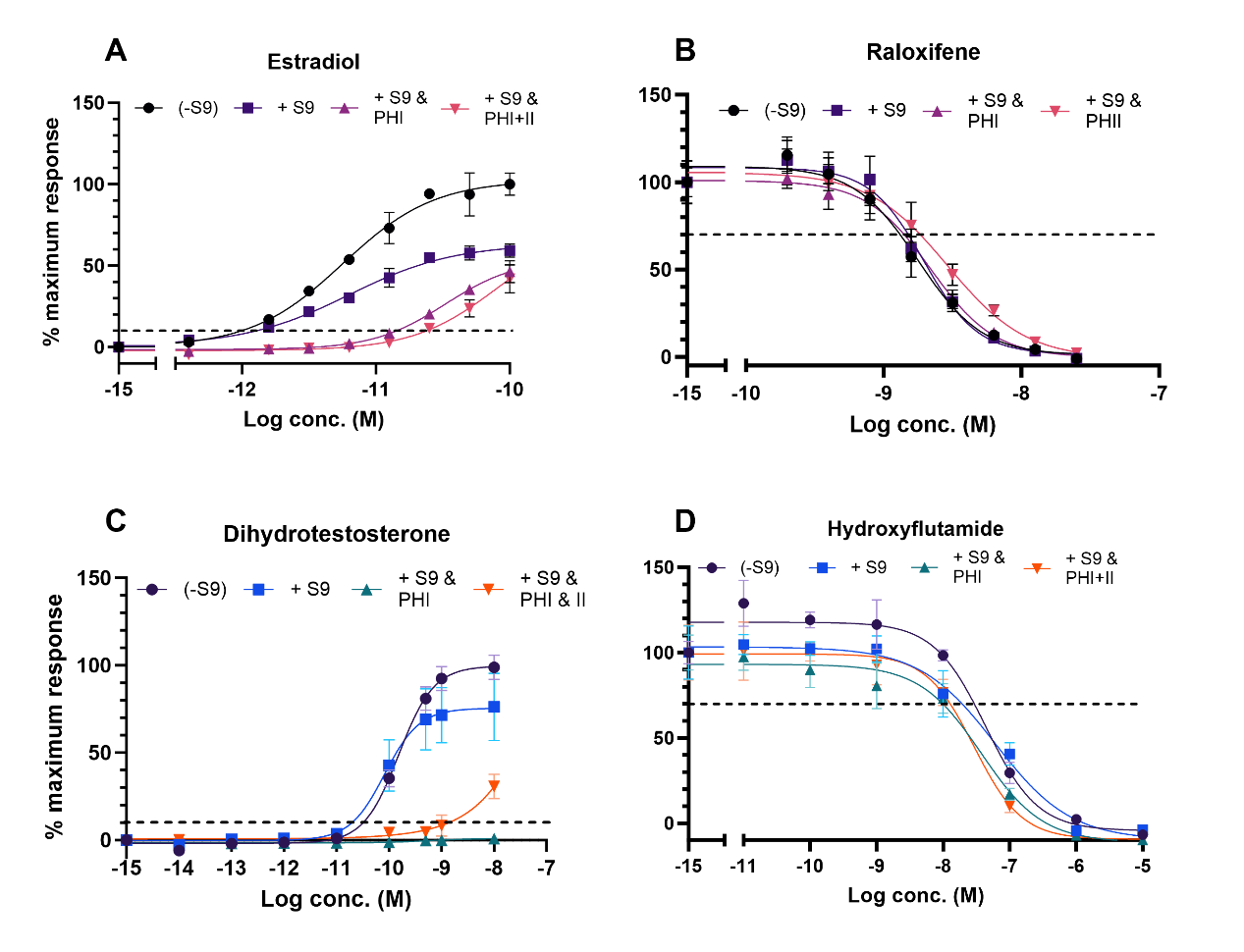


**Fig. SI-7** Concentration effect curves of ER agonistic and antagonistic activities of the reference compounds in the ER assay (A,B) and the AR assay (C,D) without and with exogenous MAS. Test concentration treatments (n=4) were normalized to vehicle controls (n=4). Data presented as mean ± SD.

1. **Incorporation of MAS into the MN assay**
   1. **Cytotoxicity of test compounds with MAS**


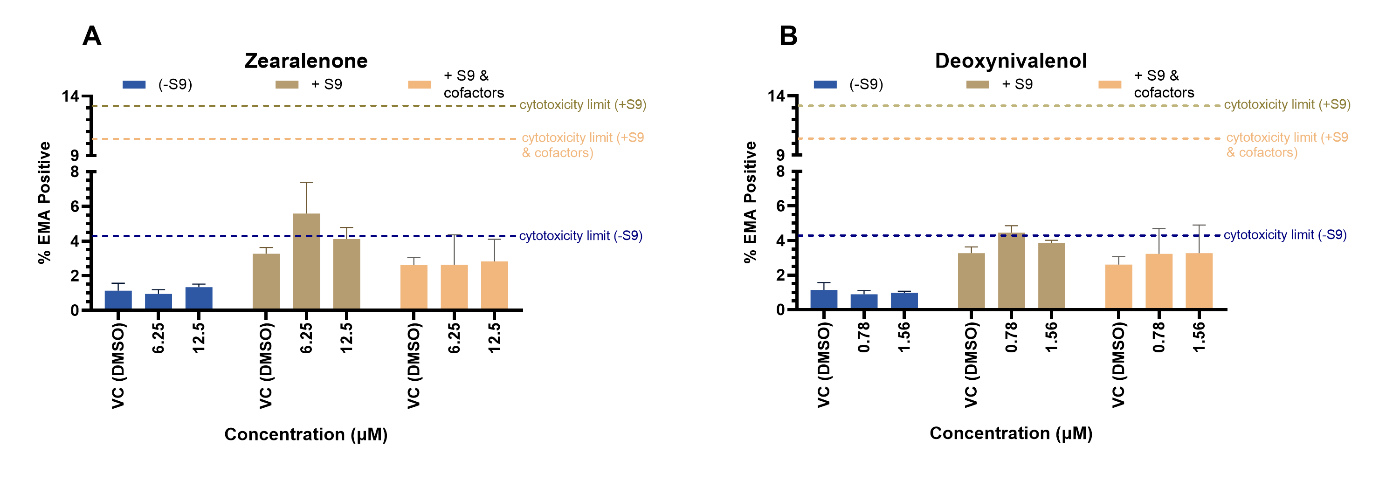


**Fig. SI-8** Cytotoxicity (expressed as % EMA-positive) in TK6 cells exposed to Zearalenone (A), and Deoxynivalenol (B). Scoring criteria for cytotoxicity limits was set as 4-fold increase in %EMA-Positive over the respective vehicle controls. Pooled treatment groups were compared to the respective limits shown as: dark blue dotted line for just test compound, dark yellow dotted line for test compound with just S9, and light orange dotted line for test compound with S9 and cofactors. Data bars for each test concentration presented as mean ± SD.
